# A diet-wide Mendelian randomization analysis: causal effects of dietary habits on type 2 diabetes

**DOI:** 10.3389/fnut.2024.1414678

**Published:** 2024-07-25

**Authors:** Rui Xiao, Li Dong, Bo Xie, Beizhong Liu

**Affiliations:** ^1^Department of General Practice, Yongchuan Hospital of Chongqing Medical University, Chongqing Medical University, Chongqing, China; ^2^Department of Nephrology and Rheumatology, Yongchuan Hospital of Chongqing Medical University, Chongqing Medical University, Chongqing, China; ^3^Central Laboratory of Yongchuan Hospital, Chongqing Medical University, Chongqing, China

**Keywords:** dietary habits, risk factors, type 2 diabetes, Mendelian randomization analysis, causal effects

## Abstract

**Background:**

Traditional clinical studies have indicated a link between certain food intakes and type 2 diabetes (T2D), but the causal relationships between different dietary habits and T2D remain unknown. Using Mendelian randomization (MR) approaches, we investigated the potential causal association between dietary habits and T2D risk.

**Methods:**

We collected publicly available genome-wide association studies’ summary statistics for 18 dietary habits from the UK Biobank and T2D data from the DIAbetes Genetics Replication And Meta-analysis (DIAGRAM) consortium. We applied the inverse variance weighted (IVW) method, supplemented with the MR-Egger method, weighted median method (WMM), simple method, weighted mode, MR-Egger regression, and the MR pleiotropy residual sum and outlier test to determine whether a particular diet was causal for T2D.

**Results:**

Reliable and robust MR estimates demonstrated that poultry intake has a causal effect on a higher risk of T2D (IVW: OR 6.30, 95% CI 3.573–11.11, *p* = 2.02e − 10; WMM: OR 5.479, 95% CI 0.2758–10.88, *p* = 1.19e − 06). Conversely, dried fruit intake (IVW: OR 0.380, 95% CI 0.237–0.608, *p* = 5.57e − 05; WMM: OR 0.450, 95% CI 0.321–0.630, *p* = 3.33e − 06) and cereal intake (IVW: OR 0.455, 95% CI 0.317–0.653, *p* = 1.924e − 05; WMM: OR 0.513, 95% CI 0.379–0.694, *p* = 1.514e − 05) were causally associated with T2D as protective factors. Sensitivity analyses confirmed the reliability and robustness of these findings.

**Discussion:**

Our study established the causal effects of poultry intake, dried fruit intake, and cereal intake on T2D, identifying poultry intake as a risk factor and the other two as protective factors. Further research into potential mechanisms is required to validate these novel findings.

## Introduction

With the rise in obesity, the decline in physical activity, a sedentary lifestyle, and poor eating habits, type 2 diabetes (T2D) is becoming more and more prevalent worldwide. In 2015, T2D was estimated to affect 415 million people, projected to reach 642 million by 2040 (1). Diabetes significantly impacts morbidity and mortality, contributing to risks such as stroke, renal failure, leg amputation, cardiovascular illnesses, vision loss, and neuropathy (2).

Lifestyle therapies aimed at modifying diet and physical activity levels have consistently been used to reduce T2D risk in the short and long term (3). Implementing effective T2D prevention initiatives as well as early detection programs is critical to lessen the disease’s health burden (4). The proper selection of food and dietary components has been recognized to play a significant role in preventing early-onset T2D and reducing the lifelong risk of developing the disease. However, the exact types of foods that are most beneficial remain unclear.

Previous studies have identified various relationships between different dietary habits and T2D. For instance, no statistically significant associations were found between the consumption of eggs, fish, nuts, vegetables, or refined grains and T2D. Conversely, dairy products, fruits, and whole grains showed a negative correlation with the incidence of T2D, while red and processed meats, as well as sugar-sweetened beverages, exhibited a positive correlation (5–10).

Dietary fiber has been shown to play a role in the etiology of chronic diseases, including type 2 diabetes (T2D), with its effects potentially mediated through the modulation of gut microbiota, making this a growing topic of research and interest (2).

While specific food risk factors for T2D progression have been identified in a few studies, there is inadequate evidence to substantiate their causal significance in T2D incidence, and the variety of diets examined is limited. Several genome-wide association studies (GWAS) have recently revealed that dietary habits are heritable features (11, 12). Therefore, Mendelian randomization (MR), which utilizes genetic instruments to mitigate potential confounding biases, is an appropriate study design to investigate the effects of nutrition on disease or health outcomes (13). The goal of this study is to use a two-sample MR technique to evaluate the causal links between eating habits and the risk of T2D. To mitigate selective reporting bias, all available dietary habit variables from the UK Biobank (UKBB) GWAS data provided by the Neale lab were evaluated for their potential causal association with T2D in this exploratory investigation.

## Methods

### Dietary habits and T2D summary statistics

Table 1 shows a brief description of dietary habits data sources. GWAS summary statistics of 18 kinds of dietary habits were from the MRC-IEU consortium and UK-Biobank, all participants are European (14, 15). UK Biobank database, a population-scale prospective cohort in the United Kingdom, >500,000 participants aged 40–69 years from 2006 to 2010 were included.

**Table 1:** Summary of dietary habits.

Exposure	GWAS data	Nsnp	Sample	R2	F
Alcohol intake frequency	ukb-b-5779	93	462,346	0.0052	55
Tea intake	ukb-b-6066	40	447,485	0.0023	62.7
Coffee intake	ukb-b-5237	38	428,860	0.0027	77
Water intake	ukb-b-14898	40	427,588	0.0017	49.9
Processed meat intake	ukb-b-6324	23	461,981	0.00078	38.6
Poultry intake	ukb-b-8006	7	461,900	0.0003	37.2
Beef intake	ukb-b-2862	15	461,053	0.00061	47.1
Pork intake	ukb-b-5640	14	460,162	0.00039	37.7
Lamb/mutton intake	ukb-b-14179	31	460,006	0.0012	41
Non-oily fish intake	ukb-b-17627	11	460,880	0.00039	44.8
Oily fish intake	ukb-b-2209	59	460,443	0.0024	46.6
Cooked vegetable intake	ukb-b-8089	17	448,651	0.00062	37.6
Salad/raw vegetable intake	ukb-b-1996	18	435,435	0.00078	44.7
Fresh fruit intake	ukb-b-3881	51	446,462	0.0023	47.6
Dried fruit intake	ukb-b-16576	41	421,764	0.0018	44.1
Cheese intake	ukb-b-1489	63	451,486	0.0018	39.8
Bread intake	ukb-b-11348	29	452,236	0.0012	45
Cereal intake	ukb-b-15926	39	441,640	0.002	49.9

For T2D, we downloaded the 2022 summary statistics of T2D in the European population from the DIAbetes Genetics Replication And Meta-analysis (DIAGRAM) consortium which is combined with a lot of T2D researchers, larger scale studies were performed to explore genetic characteristics of T2D (16). For this summary statistics, 122 GWAS including 180,834 T2D cases and 1,159,055 controls from 5 ancestry groups were used, and we only applied the summary statistics of European ancestry with 51.1% of the total effective sample size in this study (17).

### Genetic instrumental variables selection

To ensure the reliability of the instrumental variables, several statistical control steps were taken. Firstly, only single nucleotide polymorphism (SNP) with genome-wide significance ( $P<5\times 10^{-8}$ ) were extracted from the whole dataset. Secondly, linkage disequilibrium was removed using the clumping process ( $R^{2}<0.001$ , window size 10,000 kb) with the European population. Thirdly, SNPs were eliminated if minor allele frequency (MAF) < 0.01.

F statistic was calculated for every single instrumental variable to ensure there was no weak instrumental variable basis. The calculation formula is $F=R^{2}\left(N-K-1\right)/K\left(1-R^{2}\right)$ ; $R^{2}$ is the proportion of the variation for instrumental variable; N is sample size; K is the number of instrumental variables. The likelihood of a weak instrumental variable is considered very small if the F statistic is greater than 10 (18).

### Mendelian randomization analysis

The IVW method was chosen as the primary approach in this study due to its ability to aggregate Wald ratios from individual SNPs, allowing for an exploration of the causal effect between dietary habits and type 2 diabetes (T2D), provided there is minimal horizontal pleiotropy (19). On the other hand, heterogeneity effects can be accounted for in the random effects model of the IVW method (19, 20). In the MR-Egger method, we account for the presence of an intercept term and use it to assess pleiotropic effects. If the intercepted item is very close to 0, then the MR-Egger regression model is very close to IVW, but if the intercepted item is very different from 0, it means that there may be horizontal pleiotropic effects among these IVs (21). The weighted median method can produce accurate estimates even if 50% of the genetic variation violates the core assumptions of MR (22).

### Sensitivity analysis

MR Egger regression was applied to detect horizontal pleiotropy by testing the intercept (21). MR-PRESSO method was applied to detect outliers that related to horizontal pleiotropy; horizontal pleiotropy was removed by eliminating the outliers; a distortion test was used to test whether there was a significant difference before and after removing outliers (23, 24). And, heterogeneity was tested by Cochran’s Q test (IVW and MR-Egger, P<0.05 means heterogeneity existing) (24). Finally, we performed a “leave-one-out” analysis to identify the potential influential SNPs.

### Bonferroni correction

P<0.05 was considered statistically significant in MR analysis. After Bonferroni correction, P<0.0028 was considered significant (18 exposures) in this MR study.

## Results

**Figure 1:**
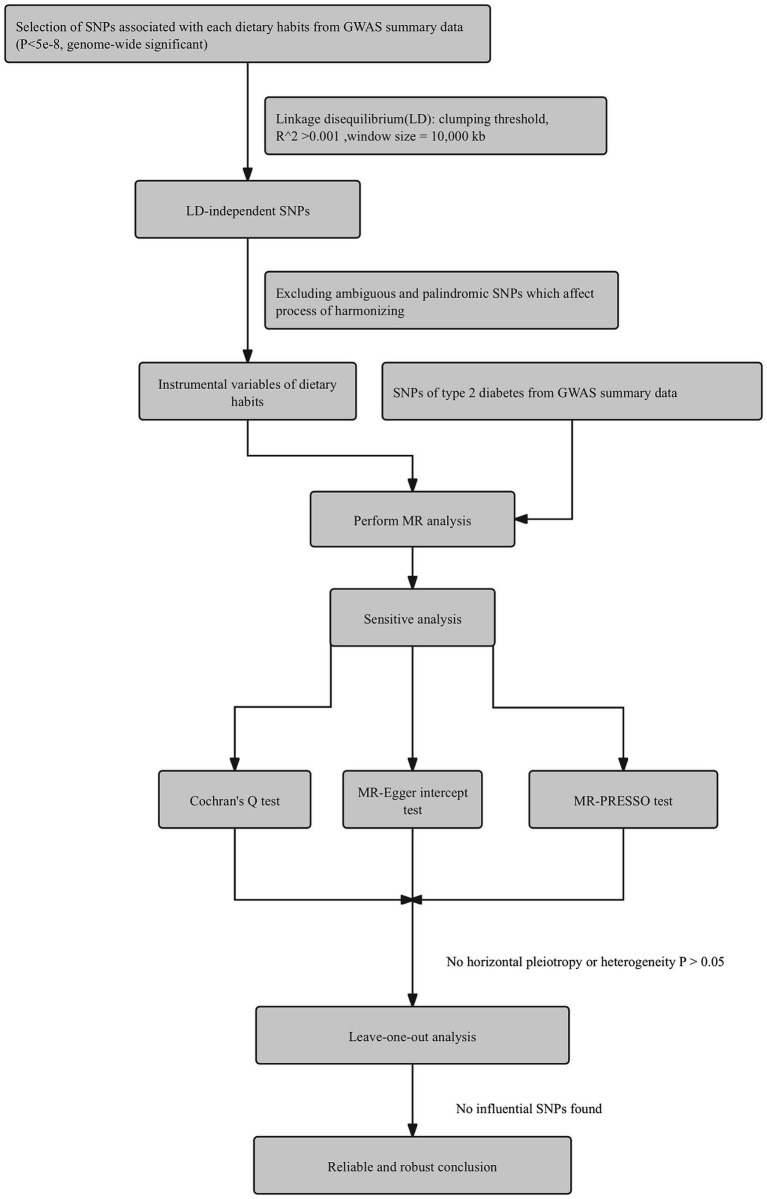
Flow chart of steps for Mendelian randomization analysis in this study.

Figure 1 shows our study flow chart. Table 2 shows the MR estimates from different methods of detecting the casual association of 18 kinds of dietary habits on T2D. Sensitivity analysis results are presented in Supplementary Table S1. According to the MR results, coffee intake, poultry intake, dried fruit intake, cheese intake, and cereal intake are genetically associated with T2D. Scatter plots, forest plots, “leave-one-out” analysis plots, and funnel plots for all 18 kinds of dietary habits are shown in Supplementary Figure S1.

**Table 2:** MR analysis results of different methods for evaluating the causality between dietary habits and T2D.

Exposures	IVW		MR-Egger		Weighted Median	
	OR (95%CI)	*p*-value	OR (95%CI)	*p*-value	OR (95%CI)	*p*-value
Alcohol intake frequency	1.325	0.004	0.664	0.05	1.070	0.31
	1.095–1.604		0.442–0.999		0.939–1.220	
Tea intake	0.943	0.789	2.150	0.12	1.539	0.0000518
	0.612–1.452		0.831–5.560		1.249–1.896	
Coffee intake	2.878	1.546e − 04	2.485	0.119	1.724	5.006e − 06
	1.664–4.975		0.813–7.591		1.364–2.178	
Water intake	1.276	0.416	0.311	0.17	0.958	0.79
	0.061–2.301		0.061–1.584		0.697–1.584	
Processed meat intake	0.955	0.852	0.105	0.06	1.032	0.85
	0.591–1.544		0.011–0.970		0.736–1.448	
Poultry intake	6.300	2.02e − 10	0.311	0.90	5.479	1.19e − 06
	3.573–1.111e+01		3.905e − 09 − 2.478e + 07		2.758e + 00 – 1.088e + 01	
Beef intake	0.936	0.94	1.031	1.00	1.345	0.300
	1.576e-01-5.564		5.795e − 05 − 18326.656		7.680e − 01 − 2.356	
Pork intake	2.106	0.115	16.000	0.42	1.980	0.035
	0.833–5.323		0.025–10148.570		1.049–3.737	
Lamb/mutton intake	0.750	0.350	5.309	0.189	0.697	0.085
	0.411–1.370		0.466–60.455		0.462–1.052	
Non-oily fish intake	1.861	0.60	69.188	0.48	0.841	0.534
	0.178–1.937e + 01		0.0007808 − 6.130721e + 06		0.488–1.451e + 00	
Oily fish intake	0.847	0.46	1.952	0.47	0.730	0.0075
	0.545–1.317		0.315–12.075		0.580–0.920	
Cooked vegetable intake	3.466	0.11	4.867	0.86	1.338	0.32
	7.439e-01-1.615e+01		1.268e − 07 − 1.868 + 08		7.554e − 01 − 2.369e + 00	
Salad/raw vegetable intake	0.636	0.05	1.616	0.69	0.627	0.071
	0.404–1.001		0.160–16.296		0.378–1.041	
Fresh fruit	1.466	0.15	3.028	0.24	1.151	0.50
intake	0.874–2.461		0.494–18.575		0.768–1.725	
Dried fruit intake	0.380	5.57e − 05	0.117	5.43e − 02	0.450	3.33e − 06
	0.237–0.608		0.014–0.974		0.321–0.630	
Cheese intake	0.452	1.72e − 12	1.163	7.43e − 01	0.572	6.46e − 07
	0.362–0.563		0.473–2.855		0.459–0.712	
Bread intake	0.872	0.61	0.810	0.87	0.837	0.33
	0.511–1.487		0.062–10.620		0.584–1.200	
Cereal intake	0.455	1.924e − 05	0.221	6.665e − 02	0.513	1.514e − 05
	0.317–0.653		0.046–1.058		0.379–0.694	

### Two-sample MR analysis for causality of coffee intake and T2D

Coffee intake was considered as a risk factor and genetically associated with increasing risk of T2D (IVW: OR, 2.878, 95%CI, 1.664–4.975, *p* = 1.546e − 04; WMM: OR, 1.724, 95%CI, 1.364–2.178, *p* = 5.006e − 06). Heterogeneity tests showed there was obvious heterogeneity (IVW, Cochran’s Q test, *p* = 2.187e-84; MR-Egger, Cochran’s Q test *p* = 1.029e − 84). No horizontal pleiotropy was found (*p* = 0.768 for the MR-Egger intercept test). Based on the MR-PRESSO method, outliers were removed to reduce heterogeneity and horizontal pleiotropy, but the correction result was distorted (*p* < 2e − 04). Thus, we cannot get a reliable and robust causal effect between coffee intake and T2D.

### Two-sample MR analysis for causality of poultry intake and T2D

**Figure 2:**
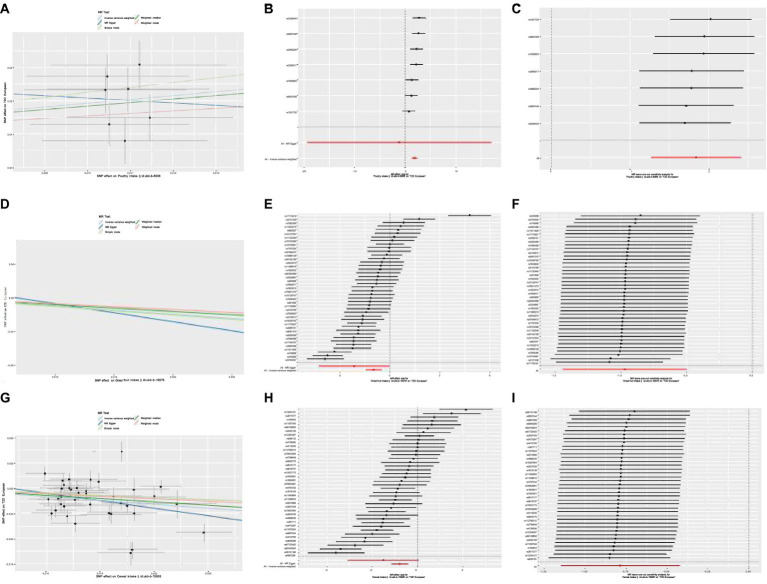
**(A)** Scatter plot showing the effect of SNPs on Poultry intake vs. T2D. **(B)** Forest plot of Mendelian randomization effect size for Poultry intake on T2D. **(C)** Leave-one-out analysis of the effect of Poultry intake on T2D. **(D)** Scatter plot showing the effect of SNPs on Dried fruit intake vs. T2D. **(E)** Forest plot of Mendelian randomization effect size for Dried fruit intake on T2D. **(F)** Leave-one-out analysis of the effect of Dried fruit on T2D. **(G)** Scatter plot showing the effect of SNPs on Cereal intake vs. T2D. **(H)** Forest plot of Mendelian randomization effect size for Cereal intake on T2D. **(I)** Leave-one-out analysis of the effect of Cereal intake on T2D.

Poultry intake was related to a higher risk of T2D according to the MR estimate results (IVW: OR,6.30,95%CI,3.573–11.11, *p* = 2.02e-10; WMM: OR,5.479,95%CI,0.2758–10.88, *p* = 1.19e-06). Heterogeneity and horizontal pleiotropy were not significant in sensitive analysis (IVW, Cochran’s Q test, *p* = 0.172; MR-Egger, Cochran’s Q test, *p* = 0.115; MR-Egger intercept test: *p* = 0.756). MR-PRESSO method did not find outliers and no influential SNPs in the “leave-one-out” analysis (Figure 2). We can get a robust and reliable causal association genetically between poultry intake and T2D that poultry intake was a risk factor for T2D.

### Two-sample MR analysis for causality of dried fruit intake and T2D

Dried fruit intake decreased the risk of T2D genetically (IVW: OR,0.380,95%CI,0.237–0.608, *p* = 5.57e − 05; WMM: OR,0.450,95%CI,0.321–0.630, *p* = 3.33e − 06). The existence of heterogeneity had been proved in heterogeneity tests (IVW, Cochran’s Q test, *p* = 3.523e − 36; MR-Egger, Cochran’s Q test, *p* = 4.939e − 35); no significant evidence of horizontal pleiotropy existed in (MR-Egger intercept close to 0). Outliers were removed in the MR-PRESSO test and there were no significant differences in effect association estimation after correction (*p* = 0.69). “Leave-one-out” analysis did not suggest any influential SNPs (Figure 2). A reliable causal relationship between dried fruit intake and T2D can be drawn.

### Two-sample MR analysis for causality of cheese intake and T2D

Cheese intake was associated with T2D genetically (IVW: OR,0.452,95%CI,0.362–0.563, *p* = 1.72e − 12; WMM: OR,0.572,95%CI,0.459–0.712, *p* = 6.46e − 07). However, there was significant horizontal pleiotropy in the MR-Egger intercept test (*p* = 0.038). We cannot draw firm conclusions about a causal relationship between cheese consumption and T2D.

### Two-sample MR analysis for causality of cereal intake and T2D

The results of MR analysis showed that cereal intake was closely related to T2D, and cereal intake could reduce the risk of T2D (IVW: OR,0.455,95%CI,0.317–0.653, *p* = 1.924e − 05; WMM: OR,0.513,95%CI,0.379–0.694, *p* = 1.514e − 05). The heterogeneity test demonstrated there was significant heterogeneity (IVW, Cochran’s Q test, *p* = 5.061e − 20; MR-Egger, Cochran’s Q test, *p* = 1.171e − 19). No horizontal pleiotropy was detected (MR-Egger intercept test: *p* = 0.358). The correction after removing outliers was not distorted in the MR-PRESSO test (*p* = 0.461). “Leave-one-out” suggested no influential SNPs existing (Figure 2). A reliable causal association between cereal intake and T2D can be drawn.

We conducted reverse Mendelian randomization (MR) analysis, with T2D as exposures and dietary habits as outcomes. Our findings indicated no significant association of T2D with poultry intake, dried fruit intake, and cereal intake, affirming the robustness of our previous results. Reverse MR estimates and sensitivity analysis outcomes are detailed in Supplementary Tables S2, S3.

## Discussion

In this two-sample MR, we systemically evaluated the causal associations of 18 dietary habits with T2D using the most extensive diet GWAS results accessible to date. Our study identified potential protective effects of dried fruit intake and cereal intake against T2D. Conversely, poultry intake was associated with an increased risk of T2D, while other dietary habits showed no significant effects on T2D risk. These findings remained consistent and robust following sensitivity analyses. Additionally, we observed genetic associations of T2D with coffee consumption and cheese consumption; however, definitive causal conclusions were limited due to the presence of horizontal pleiotropy.

In our study, we employed a two-sample MR design to investigate the causal association between various types of meat intake and T2D. Genetic variations served as instrumental variables for assessing meat intake. We examined five distinct exposures to meat, each representing different types: poultry, processed meat, beef, pork, lamb/mutton, and non-oily/oily fish. Previous studies that did not consistently differentiate between these types of meat may have contributed to conflicting findings. Our findings suggested a protective effect of poultry intake against T2D, whereas no significant associations were observed for processed meat, beef, pork, lamb/mutton, and non-oily/oily fish consumption in relation to T2D risk.

Based on its impact on obesity development, individuals who consume poultry generally have a lower incidence of type 2 diabetes compared to those who consume other types of meat (25). A reduced risk of type 2 diabetes was observed among individuals in Denmark when they replaced processed red meats (such as beef, veal, pork, lamb/mutton) with chicken, substituted whole or processed red meats with fish, and exchanged processed red meats for unprocessed red meats (26). Diabetes and poultry consumption were not significantly correlated in the Chinese cohort study of the China Kadoorie Biobank (27). These differences may be due to the mean consumption of poultry in the current study population being significantly lower than that of red meat (28). A meta-analysis of 12 different dietary groups was carried out and found a positive relationship between red, processed meat and the risk of T2D (10, 29, 30). However, in Japanese, higher red meat intake is connected with an increased risk of T2D in males but not in women (31). In Chinese adults, especially in urban participants, intake of red meat is linked to a higher risk of T2D and cardiometabolic illnesses (27). Unlike previous studies, we did a more detailed classification of red meat, for example, beef, pork, and lamb/mutton, and found that there was no significant relationship between red meat and T2D. Possible reasons include the breeding process, where commercially bred chickens may be exposed to hormones and antibiotics, which can act as endocrine disruptors and potentially affect glucose metabolism. Additionally, processed chicken products often contain high levels of sodium and preservatives. Cooking methods also play a role, as fried or fat-added chicken products can be high in saturated and trans fats. These cooking methods can lead to the formation of advanced glycation end products (AGEs) through frying, grilling, and high-temperature baking. These fats and compounds are known to increase oxidative stress and inflammation, which are contributing factors to the onset and progression of type 2 diabetes (T2D) (32). Therefore, both the nutritional and non-nutritional components of poultry may have dual effects on human health, potentially influencing outcomes positively or negatively. Different studies have yielded inconclusive results regarding the impact of meat consumption on T2D, which could be attributed to differences in study designs and methodologies, such as variations in food frequency surveys and varying levels of residual confounding. These differences may also reflect potential distinctions between Eastern and Western dietary habits.

Fish intake and the risk of T2D remain controversial. In our study, we did not find a causal relationship between non-oily/oily fish and T2D. However, some studies have reported a positive association between fish intake and T2D risk. Fish intake has been found to be positively correlated with diabetes risk, particularly among urban participants, and with cardiometabolic illnesses in Chinese adults (27). However, Some research results do not support the fish for the beneficial effect of T2D (33, 34). There was also a study that found fish/seafood and marine LC n-3 PUFA intake had no significant impact on the risk of T2D, whereas oily fish intake had a significant impact (35). The varying research results may be attributed to several factors: the potential influence of dietary toxins in fish on the association with T2D (36), differences in the types of fish consumed (lean, fatty, and shellfish), variations in dietary factors found in fish, differences in preparation methods, and varying levels of contamination across different countries (8, 37).

Wholegrain cereals have been reported to alleviate metabolic symptoms associated with T2D, including insulin resistance, lipid disorders, and obesity, by improving oxidative status, inflammatory markers, and gut microbiota (38–41). The current study provided robust evidence that cereal intake may reduce the risk of T2D, consistent with findings from previous research (42). However, both the bioactive components in cereal and the processing affect the glycemic response (38). The anti-diabetic properties of whole grains synergistically leverage their distinct bioactivities rather than relying on any single component (38). More research is needed to confirm this concept. Although specific information on cereal kinds, such as bran, oat, biscuit, and other kinds, was available in UK Biobank, GWAS summary statistics of these particular cereal types were all underpowered and found very few or no significant variants (43). As a result, we were unable to do MR analysis to determine the effect of different cereal kinds on the risk of T2D because reliable IVs were unavailable.

Previous studies found a higher diet of specific whole fruits, particularly blueberries, grapes, and apples, is connected with a significantly lower risk of type 2 diabetes (6, 44, 45). Conversely, greater consumption of fruit juice is linked with a higher risk (46, 47). However, evidence suggests a non-linear dose–response relationship; increasing fruit intake to 200–300 g/day reduces the risk of T2D by 10%, with no additional benefit observed beyond this level (10). In our study, we observed a significant association between dried fruit intake and T2D risk. Traditional dried fruits (i.e., those without added sugar), such as grapes, have been shown to reduce the risk of diabetes, likely due to the modulation of insulin resistance status by grape polyphenol extracts (48–51).

It has been suggested by some evidence that intakes of coffee, tea, and plain water were inversely associated with glucose and obesity (52–55), but several other studies have failed to replicate these associations (54, 56, 57). Confounding factors, such as differences in study design, methods of consumption quantification, beverage temperatures, and cigarette smoking, as well as variations in genetic and environmental factors such as race, sex, age, lifestyle, gut microbiota, and genetic polymorphisms, could potentially explain these discrepancies (56).

MR study, a carefully designed study with a large sample size, could help resolve this controversial issue. This is consistent with the results of other studies (13, 58). The inverse relationship between genetically proxied coffee consumption and plasma caffeine levels (i.e., the genetic variants with the strongest association with higher coffee consumption are associated with lower plasma caffeine levels) and the lack of association (e.g., from fat mass or other hot beverages or caffeine-containing drinks) may be caused by pleiotropic effects of the SNPs used (59, 60). We found no significant causal association between alcohol frequency intake, tea intake, coffee intake water intake, and T2D.

There are some limitations of our study. Firstly, due to the limitations of publicly accessible GWAS summary statistics, we only assessed the overall effects of food on T2D that were adjusted for sex and age. Secondly, the current study only included people of European ancestry, the findings may not be generalizable to other populations. Furthermore, some specific dietary patterns, for example, Mediterranean diet and Western diet, were not studied in our study, due to a lack of data. A subsample of UKBB was given repeated dietary evaluations every 3–4 months, and the results demonstrated moderate to substantial agreement with the answers to the dietary touchscreen questions at baseline (43). Thus, recall bias and seasonal variation could be ruled out.

However, this study represents one of the most comprehensive MR investigations to date into the causal role of dietary habits on the risk of T2D. To enhance statistical power, we utilized summary statistics from large-scale GWAS meta-analyses. Furthermore, sensitivity analyses demonstrated the reliability and robustness of our findings. Importantly, the primary exposure variables in our study exhibited good reproducibility and validity. Identifying protective dietary habits for T2D is critical for primary prevention. However, caution is warranted in interpreting the evidence of causality from our study. We emphasize the necessity for further research to validate and generalize our findings across different populations and settings.

Nutritional advice should be tailored to individual circumstances, including socioeconomic status, cultural background, personal preferences, and health conditions. For instance, adopting a low-carbohydrate, high-fiber diet can enhance glycemic control and optimize the effectiveness of medications like metformin and insulin (61). Certain foods rich in specific nutrients can interact with medications; for instance, foods high in vitamin K can influence the efficacy of anticoagulants used by diabetics with cardiovascular disease (62) Additionally, dietary patterns that facilitate weight loss can enhance insulin sensitivity, potentially reducing the required dosage of medications (63). Therefore, consulting with a certified dietitian or healthcare professional is recommended to create a personalized diet plan that aligns with individual needs and health goals.

Based on our findings, we offer the following dietary recommendations: Firstly, avoid choosing to consume poultry that has been overexposed to hormones and antibiotics during the feeding process. In addition, avoid choosing poultry that is overly processed with too much-added sodium and preservatives. Choose simple cooking methods such as baking or steaming and avoid frying and grilling (32). Secondly, moderate intake of dried fruits with simple ingredients is recommended in daily life; many dried fruits are rich in antioxidant vitamins, and the dietary fiber in dried fruits modulates gut microbiology and influences lipid and glucose metabolism and immune homeostasis (48, 64–66). Finally, in terms of staple foods in the diet, choosing the right amount of cereals—typically low in added sugars and high in fiber—can be beneficial in preventing T2D (67). Most importantly, adopting a combination of low-risk lifestyle behaviors—including maintaining a healthy weight, consuming a balanced diet, engaging in regular physical activity, refraining from smoking or excessive alcohol consumption, and drinking alcohol in moderation—has been associated with a lower risk of developing type 2 diabetes (68).

## Conclusion

This two-sample MR study demonstrated a causal effect of poultry intake, dried fruit intake, and cereal intake on T2D. Specifically, poultry intake was identified as a genetic risk factor for T2D, whereas dried fruit intake and cereal intake were found to be protective factors. Further validation of these novel findings and investigation into potential underlying mechanisms are warranted.

## Data availability statement

The original contributions presented in the study are included in the article/Supplementary material, further inquiries can be directed to the corresponding author.

## Ethics statement

The studies involving humans were approved by All GWAS summary data came from a public database, no raw data was used for this study. UK Biobank has approval from the North West Multi-centre Research Ethics Committee (MREC) as a Research Tissue Bank (RTB) approval, detailed information can be found in 241 https://www.ukbiobank.ac.uk/learn-more-about-uk-biobank/about-us/ethics. DIAGRAM Consortium is available from: http://www.diagram-consortium.org/pub.html. Thus, there are no ethical problems in this article. The studies were conducted in accordance with the local legislation and institutional requirements. The participants provided their written informed consent to participate in this study.

## Author contributions

RX: Conceptualization, Methodology, Software, Writing – original draft. LD: Investigation, Visualization, Writing – review & editing. BX: Supervision, Writing – review & editing. BL: Supervision, Writing – review & editing.
