# Supplementary material for: A diet-wide Mendelian randomization analysis: causal effects of dietary habits on type 2 diabetes
Source: Front Nutr. 2024 Jul 25;11:1414678. doi: 10.3389/fnut.2024.1414678 (PMC11306177; doi:10.3389/fnut.2024.1414678)
Supplement: Supplementary file 1 [file Table_1.docx]

**Table S1** Sensitivity analysis of dietary habits and T2D.

| **Exposure** | **Heterogeneity** | | | | **Test for directional horizontal pleiotropy** | | | | | | |
| --- | --- | --- | --- | --- | --- | --- | --- | --- | --- | --- | --- |
|  | IVW | | MR Egger | | Egger_intercept | se | pval | MR-PRESSO  global | MR-PRESSO  distortion | MR-PRESSO  Outlier test | |
|  | Q | Q_pval | Q | Q_pval |  |  |  | pval | pval | pval | Outliers |
| Alcohol intake frequency | 938.411  (92) | 2.535e-140 | 815.798  (91) | 6.629e-117 | 0.0194 | 0.0052 | 0.0003709828 | <2e-04 | 0.1078 | <0.0192 | 3(rs11039429) 10(rs11940694)  13(rs1229984)  19(rs1421085)  30(rs2160935)  38(rs28768122)  44(rs34631026)  46(rs35105141)  47(rs362307)  57(rs4940926)  65(rs62305780)  68(rs650558)  83(rs780094)  88(rs8614)  96(rs9912298) |
| Tea intake | 499.043  (39) | 3.725e-81 | 456.000  (38) | 4.496e-73 | -0.01716371 | 0.0090626 | 0.0658687 | <2e-04 | 0.5132 | <0.0082 | 3(rs10764990)  19(rs2279844)  21(rs2472297)  26(rs4410790)  27(rs4808193)  37(rs9302428)  41(rs9937354) |
| Coffee intake | 509.429  (37) | 2.187e-84 | 508.186  (36) | 1.029e-84 | 0.002761843 | 0.009306445 | 0.7683501 | <2e-04 | <2e-04 | <0.008 | 7(rs13054099)  9(rs1338549)  10(rs13387939)  11(rs1421085)  17(rs2472297)  23(rs476828)  24(rs516636)  27(rs6062682)  30(rs62064918)  33(rs7224815)  36(rs780093)  38(rs78267637) |
| Water intake | 552.746  (39) | 5.339e-92 | 508.610  (38) | 1.198e-83 | 0.02234006 | 0.01230225 | 0.07727696 | <2e-04 | 0.334 | <0.008 | 7(rs1421085)  17(rs2472297)  26(rs429358)  27(rs4410790)  35(rs7626335)  36(rs782221) |
| Processed meat intake | 123.908  (22) | 3.386e-16 | 104.287  (21) | 4.996e-13 | 0.03367836 | 0.01694338 | 0.06004022 | <2e-04 | 0.182 | <0.0046 | 5(rs1422192)  12(rs4240672)  14(rs6010651)  19(rs7531118)  20(rs77165542) |
| Poultry intake | 9.031  (6) | 0.172 | 8.845  (5) | 0.115 | 0.03259914 | 0.1005256 | 0.7588478 | 0.3612 | NA | NA | NA |
| Beef intake | 497.642  (14) | 2.931e-97 | 497.627  (13) | 4.671e-98 | -0.001216537 | 0.06222931 | 0.9846998 | <2e-04 | 0.714 | <0.0034 | 1(rs10789340)  6(rs12247907)  8(rs1421085)  10(rs429358)  12(rs62169335)  13(rs62396185)  15(rs784251) |
| Pork intake | 79.840  (13) | 1.183e-11 | 77.341  (12) | 1.324e-11 | -0.02099551 | 0.0337153 | 0.5451149 | <2e-04 | 0.837 | <0.0028 | 1(rs10972033)  3(rs12721051)  5(rs2387807) |
| Lamb/mutton intake | 181.286  (30) | 1.473e-23 | 166.166  (29) | 3.494e-21 | -0.02190623 | 0.01348515 | 0.1150963 | <2e-04 | 0.6696 | <0.0064 | 20(rs429358)  24(rs62106258)  32(rs994270) |
| Non-oily fish intake | 446.706  (10) | 1.053e-89 | 427.464  (9) | 1.877e-86 | -0.04520415 | 0.07101881 | 0.540293 | <2e-04 | <2e-04 | <0.0022 | 2(rs1260326)  8(rs56094641)  10(rs7148387) |
| Oily fish intake | 688.958  (58) | 9.722e-109 | 678.764  (57) | 3.022e-107 | -0.01248828 | 0.01349711 | 0.3587339 | <2e-04 | 0.477 | <0.0122 | 9(rs11986122)  17(rs1421085)  20(rs1876245)  33(rs45501495)  40(rs59355765)  42(rs6059844)  47(rs703987)  61(rs9958909) |
| Cooked vegetable intake | 373.870 (16) | 1.074e-69 | 373.833 (15) | 2.148e-70 | -0.003494354 | 0.0913127 | 0.9699787 | <2e-04 | 0.0236 | <0.0034 | 2(rs10161952)  6(rs1421085)  11(rs2844672) |
| Salad/raw vegetable intake | 31.003 (17) | 0.020 | 29.789  (16) | 0.019 | -0.01000919 | 0.0123949 | 0.4312063 | 0.011 | 0.4772 | 0.021 | 17(rs7619139) |
| Fresh fruit intake | 300.279  (50) | 2.058e-37 | 296.235  (49) | 4.537e-37 | -0.006890234 | 0.008424605 | 0.4173907 | <2e-04 | 0.23 | <0.0104 | 7(rs10838724)  8(rs10840126)  13(rs12044599)  30(rs28479795)  31(rs2867113)  37(rs586346)  41(rs73455661)  44(rs7818437)  51(rs9919429)  52(rs994270) |
| Dried fruit intake | 271.497  (40) | 3.523e-36 | 263.102  (39) | 4.939e-35 | 0.01461272 | 0.01309965 | 0.271461 | <2e-04 | 0.6904 | <0.0086 | 16(rs17175518)  22(rs3101339)  24(rs3764002)  28(rs429358)  33(rs746868) |
| Cheese intake | 234.452  (62) | 7.297e-22 | 218.312  (61) | 1.473e-19 | -0.01621098 | 0.007633455 | 0.03776093 | <2e-04 | 0.1826 | <0.0128 | 4(rs10938397)  34(rs4776970)  41(rs61953351) |
| Bread intake | 240.511  (28) | 1.175e-35 | 240.482  (27) | 3.934e-36 | 0.001061791 | 0.01841493 | 0.9544447 | <2e-04 | 0.7532 | <0.0062 | 2(rs11060853)  3(rs11183201)  5(rs13016665)  13(rs28406095)  14(rs4665972)  18(rs62091167)  27(rs9529024)  28(rs9564268) |
| Cereal intake | 178.131  (38) | 5.061e-20 | 174.056  (37) | 1.171e-19 | 0.01053171 | 0.01131557 | 0.3580279 | <2e-04 | 0.4612 | <0.0086 | 11(rs13234131)  18(rs2817377)  27(rs56131196)  28(rs62442924)  43(rs9987289) |
