# Supplementary material for: A diet-wide Mendelian randomization analysis: causal effects of dietary habits on type 2 diabetes
Source: Front Nutr. 2024 Jul 25;11:1414678. doi: 10.3389/fnut.2024.1414678 (PMC11306177; doi:10.3389/fnut.2024.1414678)
Supplement: Supplementary file 2 [file Table_2.docx]

**Table S2** Reverse MR analysis results of different methods for evaluating the causality between dietary habits and T2DM

| Outcomes | IVW | | MR-Egger | | Weighted Median | |
| --- | --- | --- | --- | --- | --- | --- |
|  | ß(95%CI) | P-value | ß(95%CI) | P-value | ß(95%CI) | P-value |
| Alcohol intake frequency | 0.044  0.026-0.062 | 2.059e-06 | -0.010  -0.050-0.031 | 0.637 | 0.032  0.017-0.468 | 2.073e-05 |
| Tea intake | -0.007  -0.016-1.935e-03 | 0.127 | -0.014  -0.033-6.156e-03 | 0.178 | -0.011  -0.022-2.035e-04 | 0.054 |
| Coffee intake | -0.001  -0.010-0.008 | 0.771 | -0.005  -0.026-0.015 | 0.619 | -0.007  -0.015-0.002 | 0.114 |
| Water intake | 0.011  0.003-0.020 | 0.006 | 0.013  -0.006- -0.324 | 0.179 | 0.012  0.003-0.022 | 0.012 |
| Processed meat intake | 6.007e-03  -0.003-0.015 | 0.173 | -3.976e-03  -0.024-0.016 | 0.691 | 3.427e-03  -0.006-0.013 | 0.494 |
| Poultry intake | 9.386e-03  0.003-0.016 | 0.006 | 1.529e-03  -0.014-0.017 | 0.843 | 3.885e-03  -0.005-0.013 | 0.395 |
| Beef intake | -0.001  -0.008-0.007 | 0.874 | -0.009  -0.025-0.008 | 0.292 | 0.002  -0.007-0.011 | 0.644 |
| Pork intake | 0.006  0.001-0.011 | 0.030 | 0.001  -0.011-0.013 | 0.913 | 0.003  -0.004-0.010 | 0.387 |
| Lamb/mutton intake | -0.001  -0.007-0.005 | 0.707 | 0.003  -0.011-0.017 | 0.689 | 0.001  -0.006-0.007 | 0.879 |
| Non-oily fish intake | 0.002  -0.004-0.008 | 0.475 | 0.006  -0.008-0.019 | 0.401 | 0.005  -0.002-0.012 | 0.183 |
| Oily fish intake | -0.002  -0.010-0.006 | 0.656 | 0.004  -0.015-0.023 | 0.671 | 0.000013  -0.010-0.010 | 0.998 |
| Cooked vegetable intake | 0.005  -0.001-0.011 | 0.105 | -0.0016  -0.015-0.012 | 0.813 | 0.001  -0.006-0.009 | 0.732 |
| Salad/raw vegetable intake | 0.000313  -0.005-0.006 | 0.909 | 0.007  -0.005-0.019 | 0.252 | 0.001  -0.006-0.009 | 0.746 |
| Fresh fruit intake | 0.007  0.001-0.013 | 0.024 | 0.004  -0.009-0.017 | 0.575 | 0.004  -0.003- 0.010 | 0.236 |
| Dried fruit intake | 0.000313  -0.005-0.006 | 0.909 | 0.007  -0.005-0.019 | 0.252 | 0.001  -0.006-0.009 | 0.746 |
| Cheese intake | -0.017  -0.026- -0.007 | 0.00077 | -0.005  -0.027-0.016 | 0.625 | -0.016  -0.028- -0.004 | 0.010 |
| Bread intake | 0.008  -0.000407-0.016 | 0.063 | 0.011  -0.007-0.030 | 0.238 | 0.013  0.003-0.022 | 0.013 |
| Cereal intake | -0.001  -0.009-0.006 | 0.758 | -0.011  -0.028-0.006 | 0.211 | 0.00024  -0.008- 0.008 | 0.954 |
