# Supplementary material for: A diet-wide Mendelian randomization analysis: causal effects of dietary habits on type 2 diabetes
Source: Front Nutr. 2024 Jul 25;11:1414678. doi: 10.3389/fnut.2024.1414678 (PMC11306177; doi:10.3389/fnut.2024.1414678)
Supplement: Supplementary file 3 [file Table_3.docx]

**Table S3** Sensitivity analysis of dietary habits and T2D(Reverse MR).

| **Exposure** | **Heterogeneity** | | | | **Test for directional horizontal pleiotropy** | | | | | | |
| --- | --- | --- | --- | --- | --- | --- | --- | --- | --- | --- | --- |
|  | IVW | | MR Egger | | Egger_intercept | se | pval | MR-PRESSO  global | MR-PRESSO  distortion | MR-PRESSO  Outlier test | |
|  | Q | Q_pval | Q | Q_pval |  |  |  | pval | pval | pval | Outliers |
| Alcohol intake frequency | 964.309  (180) | 1.893e-107 | 920.839  (179) | 3.815e-100 | 0.00397981 | 0.001369086 | 0.004111744 | <2e-04 | 0.5586 | <0.0372 | 34(rs12325539)  35(rs1260326)  44(rs13389219)  70(rs1783541)  99(rs329122)  105(rs35011184)  108(rs35895680)  113(rs3798519)  121(rs474513)  133(rs55872725)  136(rs5758223)  144(rs62271373)  176(rs8008910)  178(rs8097210) |
| Tea intake | 452.347 (180) | 2.075e-25 | 450.903  (179) | 2.018e-25 | 0.0005073264 | 0.0006702285 | 0.4500777 | <2e-04 | 0.522 | <0.0372 | 41(rs13130484)  113(rs3798519)  133(rs55872725)  141(rs601945)  156(rs6937438)  164(rs739846) |
| Coffee intake | 834.780  (180) | 6.985e-85 | 833.985  (179) | 4.412e-85 | 0.0002868378 | 0.0006944446 | 0.680067 | <2e-04 | 0.5494 | <0.0372 | 35(rs1260326)  40(rs13022337)  41(rs13130484)  44(rs13389219)  99(rs329122)  110(rs3768321)  113(rs3798519)  125(rs490689)  126(rs4925109)  133(rs55872725)  143(rs62107261)  161(rs72802358)  178(rs8097210) |
| Water intake | 577.196  (180) | 3.725e-43 | 577.119  (179) | 2.122e-43 | -9.974954e-05 | 0.0006486124 | 0.8779492 | <2e-04 | 0.297 | <0.0372 | 1(rs1007090)  32(rs12001437)  34(rs12325539)  35(rs1260326)  66(rs17522122)  95(rs2972144)  115(rs429358)  133(rs55872725)  159(rs7178762)  177(rs8071043) |
| Processed meat intake | 453.074  (180) | 1.663e-25 | 449.955  (179) | 2.695e-25 | 0.0007372939 | 0.0006618336 | 0.2667655 | <2e-04 | 0.6738 | <0.0372 | 35(rs1260326)  115(rs429358)  125(rs490689)  142(rs61676547)  143(rs62107261) |
| Poultry intake | 357.055(180) | 8.826e-14 | 354.487((179) | 1.191e-13 | 0.0005802871 | 0.0005096241 | 0.2563678 | <2e-04 | 0.975 | <0.0372 | 35(rs1260326)  111(rs3783394)  119(rs4688760) |
| Beef intake | 482.138(180) | 1.979e-29 | 478.902  (179) | 3.348e-29 | 0.0006132738 | 0.0005576532 | 0.2729221 | <2e-04 | 0.9078 | <0.0372 | 50(rs1426371)  111(rs3783394)  115(rs429358)  133(rs55872725)  178(rs8097210)  184(rs9563615) |
| Pork intake | 351.192(180) | 3.853e-13 | 349.455((179) | 4.219e-13 | 0.0003874866 | 0.0004107228 | 0.3467346 | <2e-04 | 0.9598 | <0.0372 | 32(rs12001437)  115(rs429358)  125(rs490689) |
| Lamb/mutton intake | 480.215(180) | 3.635e-29 | 479.149  (179) | 3.096e-29 | -0.0003011534 | 0.0004772464 | 0.5288304 | <2e-04 | 0.8888 | <0.0372 | 115(rs429358)  143(rs62107261) |
| Non-oily fish intake | 348.642(180) | 7.261e-13 | 347.970  (179) | 6.106e-13 | -0.0002649378 | 0.0004505899 | 0.5572869 | <2e-04 | 0.232 | <0.0372 | 35(rs1260326)  104(rs34990153)  115(rs429358)  133(rs55872725) |
| Oily fish intake | 500.604(180) | 5.373e-32 | 499.26  (179) | 4.932e-32 | -0.000440849 | 0.0006367407 | 0.4896131 | <2e-04 | 0.673 | <0.0372 | 1(rs1007090)  6(rs1061810)  35(rs1260326)  41(rs13130484)  97(rs3094682)  115(rs429358)  133(rs55872725)  158(rs703972) |
| Cooked vegetable intake | 391.915(180) | 8.734e-18 | 389.468  (179) | 1.151e-17 | 0.0004867666 | 0.0004590798 | 0.2904325 | <2e-04 | 0.5626 | <0.0372 | 21(rs115505614)  97(rs3094682)  133(rs55872725)  156(rs6937438) |
| Salad/raw vegetable intake | 380.635(180) | 1.872e-16 | 377.472  (179) | 2.984e-16 | -0.0005049781 | 0.0004123392 | 0.2223101 | <2e-04 | 0.1746 | <0.0372 | 35(rs1260326)  115(rs429358)  133(rs55872725)  143(rs62107261) |
| Fresh fruit intake | 610.880(180) | 2.745e-48 | 610.087  (179) | 1.963e-48 | 0.000217027 | 0.0004497881 | 0.6300336 | <2e-04 | 0.0702 | <0.0372 | 3(rs10406431)  13(rs10974438)  34(rs12325539)  40(rs13022337)  113(rs3798519)  115(rs429358)  122(rs4804833)  143(rs62107261)  152(rs672271)  176(rs8008910)  178(rs8097210) |
| Dried fruit intake | 524.551(180) | 2.117e-35 | 520.412 (179) | 4.858e-35 | -0.0006774949 | 0.0005677905 | 0.2343651 | <2e-04 | 0.1454 | <0.0372 | 42(rs13330951)  50(rs1426371)  97(rs3094682)  110(rs3768321)  111(rs3783394)  115(rs429358)  133(rs55872725)  178(rs8097210)  184(rs9563615) |
| Cheese intake | 496.505(180) | 2.016e-31 | 493.125  (179) | 3.581e-31 | -0.0008192082 | 0.0007395622 | 0.2694805 | <2e-04 | 0.3242 | <0.0372 | 11(rs10908278)  41(rs13130484)  104(rs34990153)  115(rs429358)  119(rs4688760)  136(rs5758223) |
| Bread intake | 442.059(180) | 4.656e-24 | 441.653  (179) | 3.332e-24 | -0.0002527881 | 0.0006230294 | 0.6854181 | <2e-04 | 0.343 | <0.0372 | 4(rs10419627)  35(rs1260326)  40(rs13022337)  50(rs1426371)  80(rs2277536)  95(rs2972144)  142(rs61676547)  143(rs62107261)  178(rs8097210) |
| Cereal intake | 487.253  (180) | 3.900e-30 | 483.054  (179) | 8.971e-30 | 0.0007279186 | 0.0005835707 | 0.2138976 | <2e-04 | 0.0942 | <0.0372 | 29(rs11842871)  40(rs13022337)  115(rs429358)  129(rs505922)  178(rs8097210) |
