# Supplementary material for: A diet-wide Mendelian randomization analysis: causal effects of dietary habits on type 2 diabetes
Source: Front Nutr. 2024 Jul 25;11:1414678. doi: 10.3389/fnut.2024.1414678 (PMC11306177; doi:10.3389/fnut.2024.1414678)
Supplement: Supplementary file 4 [file Image_1.pdf]

Figure S1.1 Scatter plot showing the effects of SNPs on dietary habits on the T2D

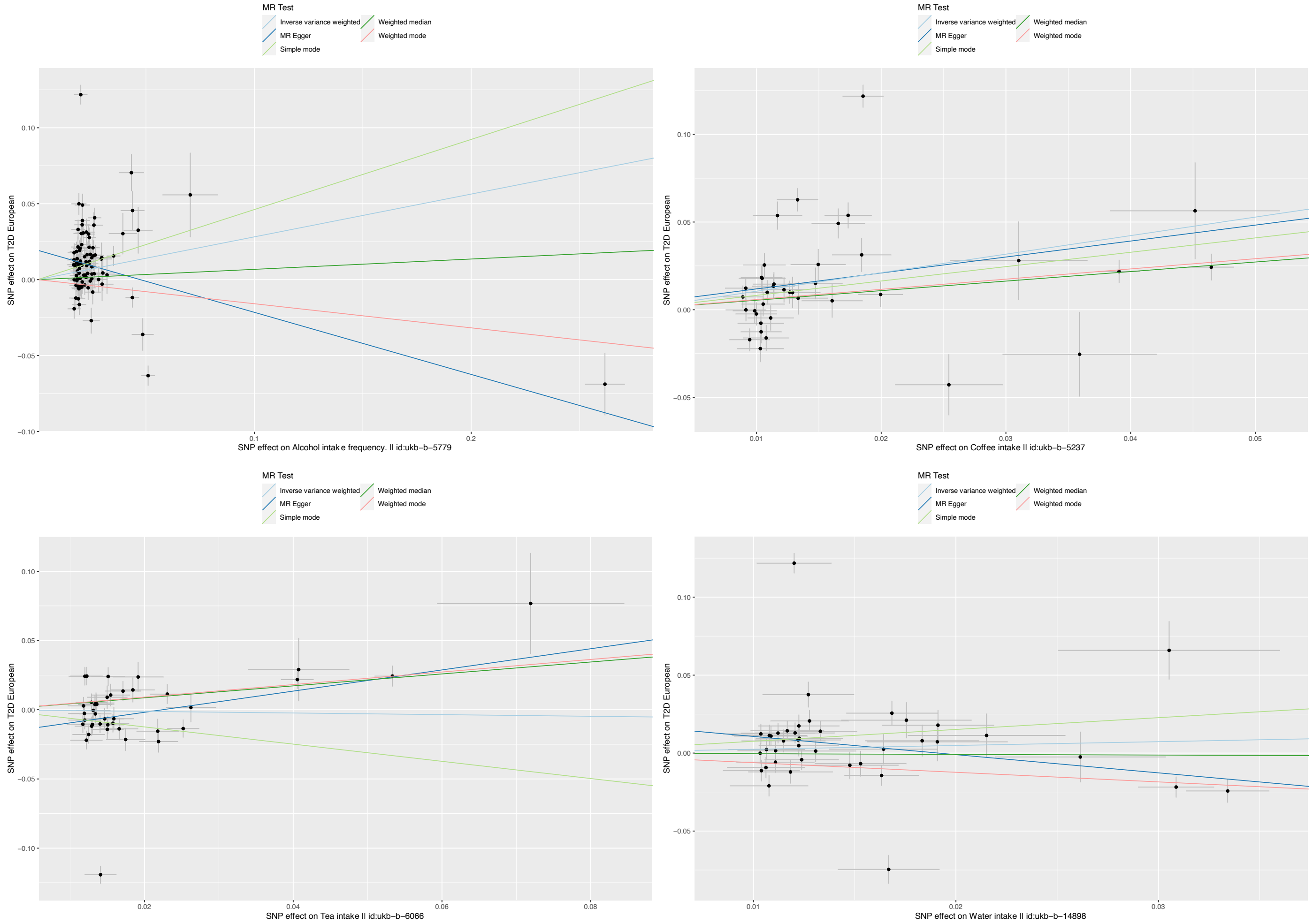

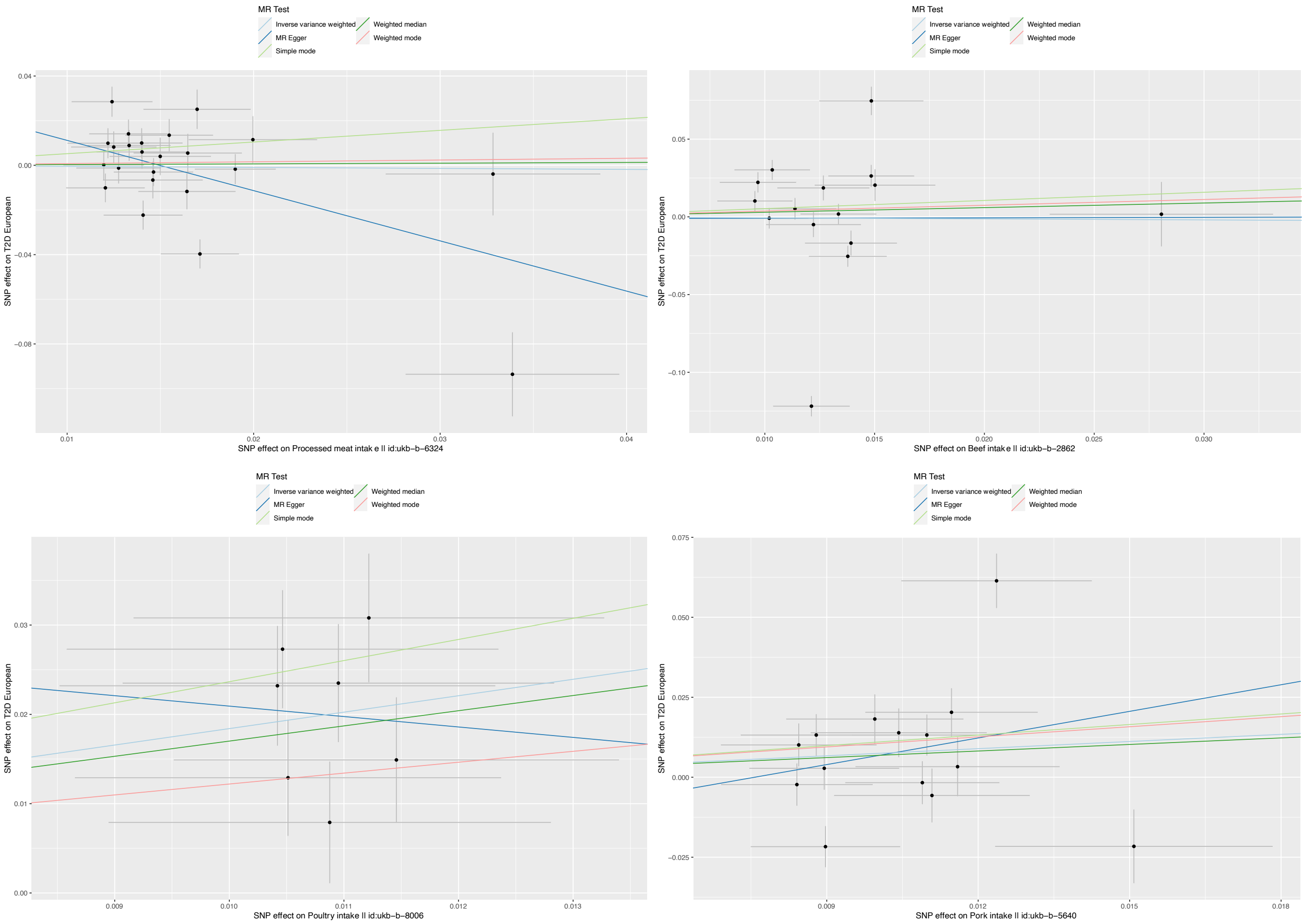
MR Test
Inverse variance weighted
MR Egger
Simple mode
Weighted median
Weighted mode
SNP effect on T2D European
SNP effect on Processed meat intak e ll id:ukb−b−6324
SNP effect on Beef intak e ll id:ukb−b−2862
SNP effect on Poultry intake ll id:ukb−b−8006
SNP effect on Pork intake ll id:ukb−b−5640

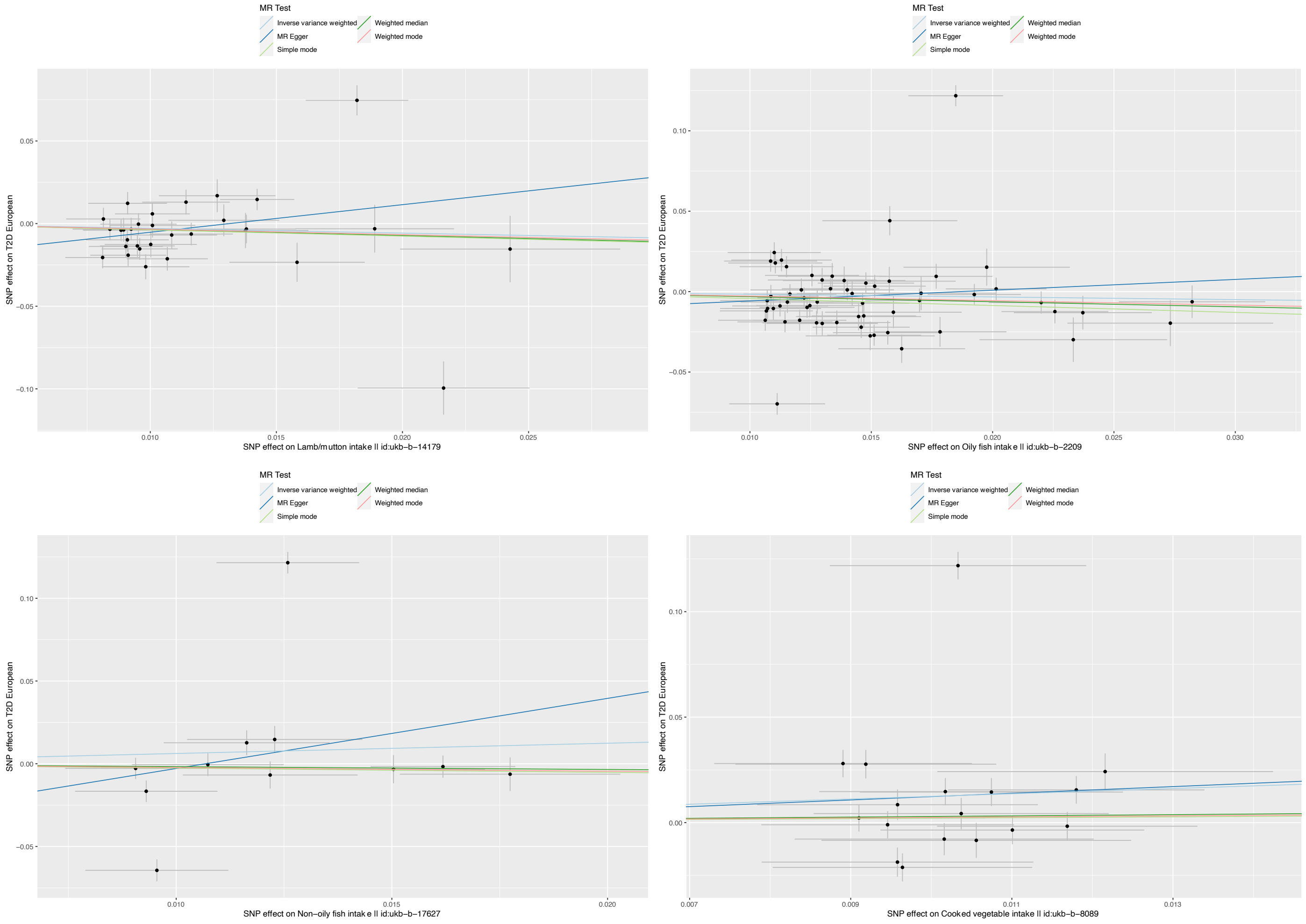
MR Test
Inverse variance weighted
MR Egger
Simple mode
Weighted median
Weighted mode
SNP effect on T2D European
SNP effect on Lamb/mutton intake || id:ukb-b-14179
SNP effect on Oily fish intake || id:ukb-b-2209
SNP effect on Non-oily fish intake || id:ukb-b-17627
SNP effect on Cooked vegetable intake || id:ukb-b-8089

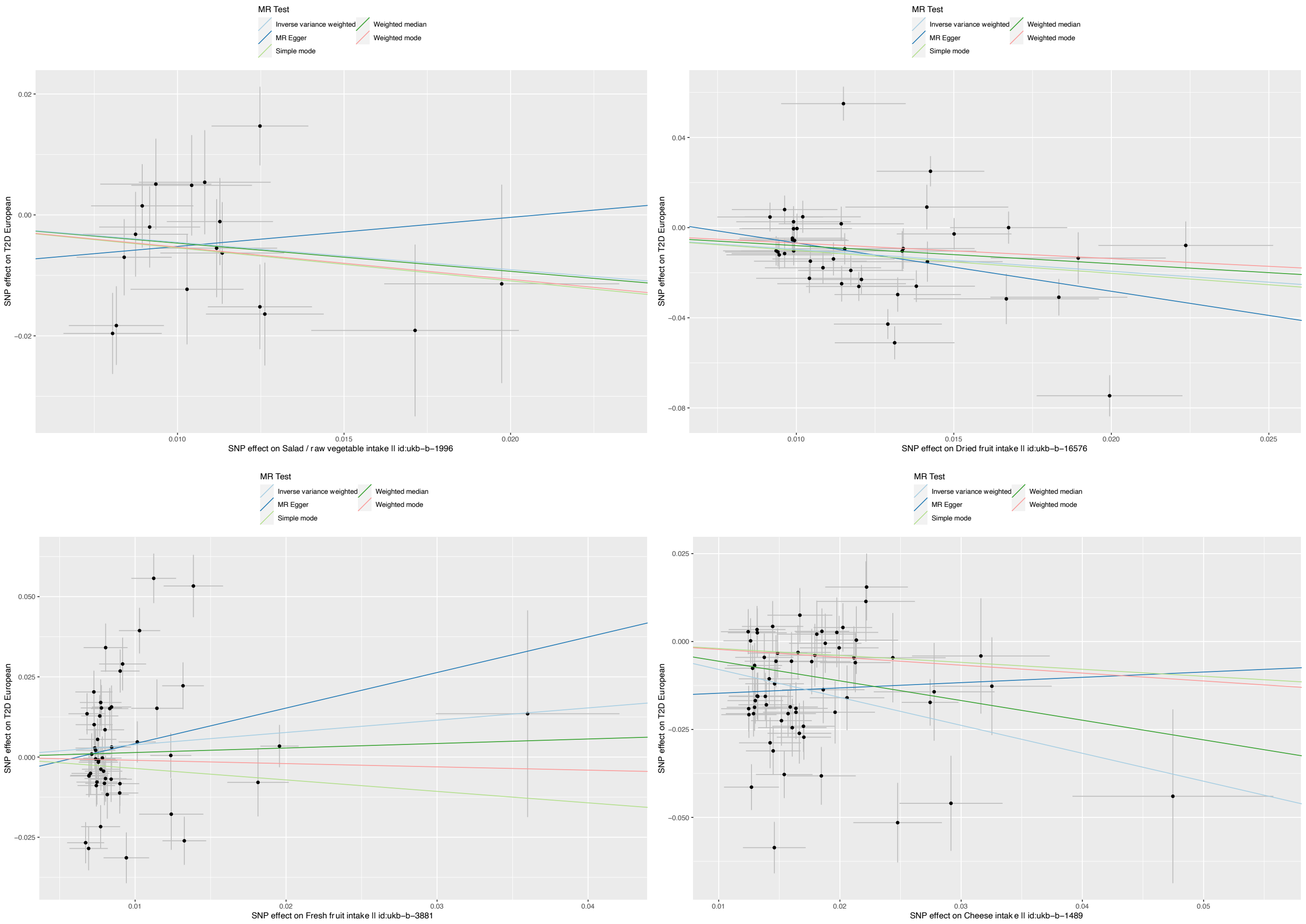

MR Test
Inverse variance weighted
MR Egger
Simple mode
Weighted median
Weighted mode
SNP effect on T2D European
SNP effect on Salad / raw vegetable intake || id:ukb-b-1996
SNP effect on Dried fruit intake || id:ukb-b-16576
SNP effect on Fresh fruit intake || id:ukb-b-3881
SNP effect on Cheese intake || id:ukb-b-1489

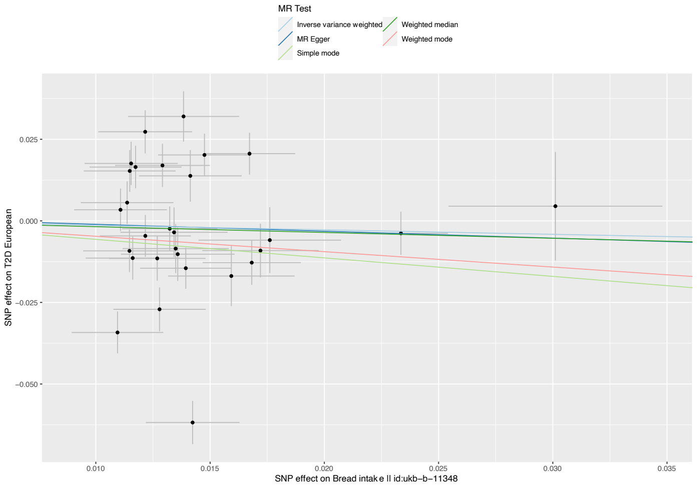

MR Test
Inverse variance weighted
MR Egger
Simple mode
Weighted median
Weighted mode
SNP effect on T2D European
SNP effect on Bread intake || id:ukb-b-11348
0.025
0.000
−0.025
−0.050
0.010
0.015
0.020
0.025
0.030
0.035

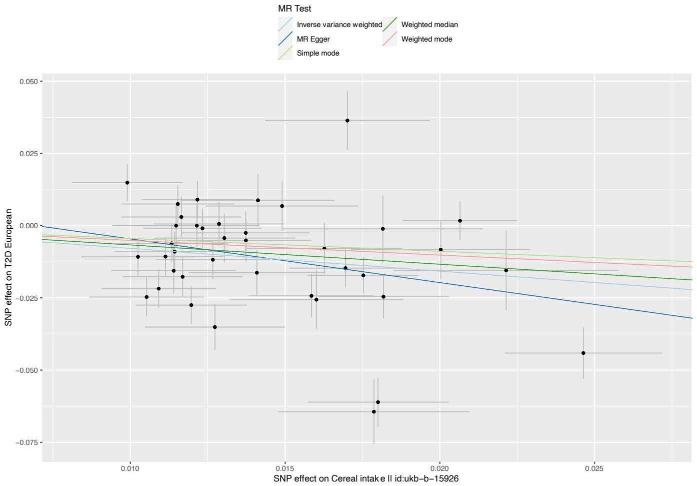

MR Test
Inverse variance weighted
MR Egger
Simple mode
Weighted median
Weighted mode
SNP effect on T2D European
SNP effect on Cereal intake || id:ukb-b-15926
0.050
0.025
0.000
−0.025
−0.050
−0.075
0.010
0.015
0.020
0.025

Figure S1.2 Leave-one-out analysis of the effect of dietary habits on T2D

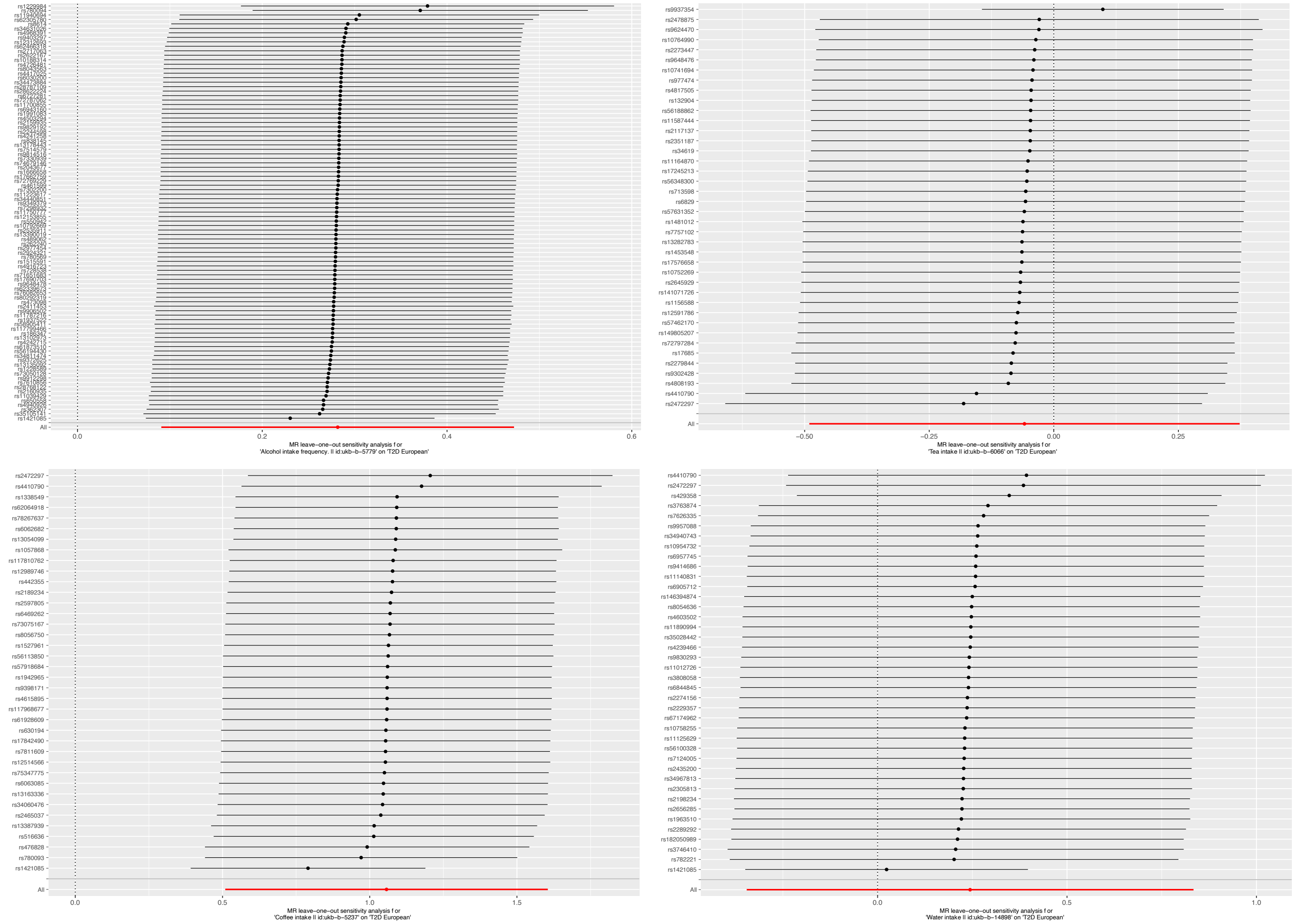

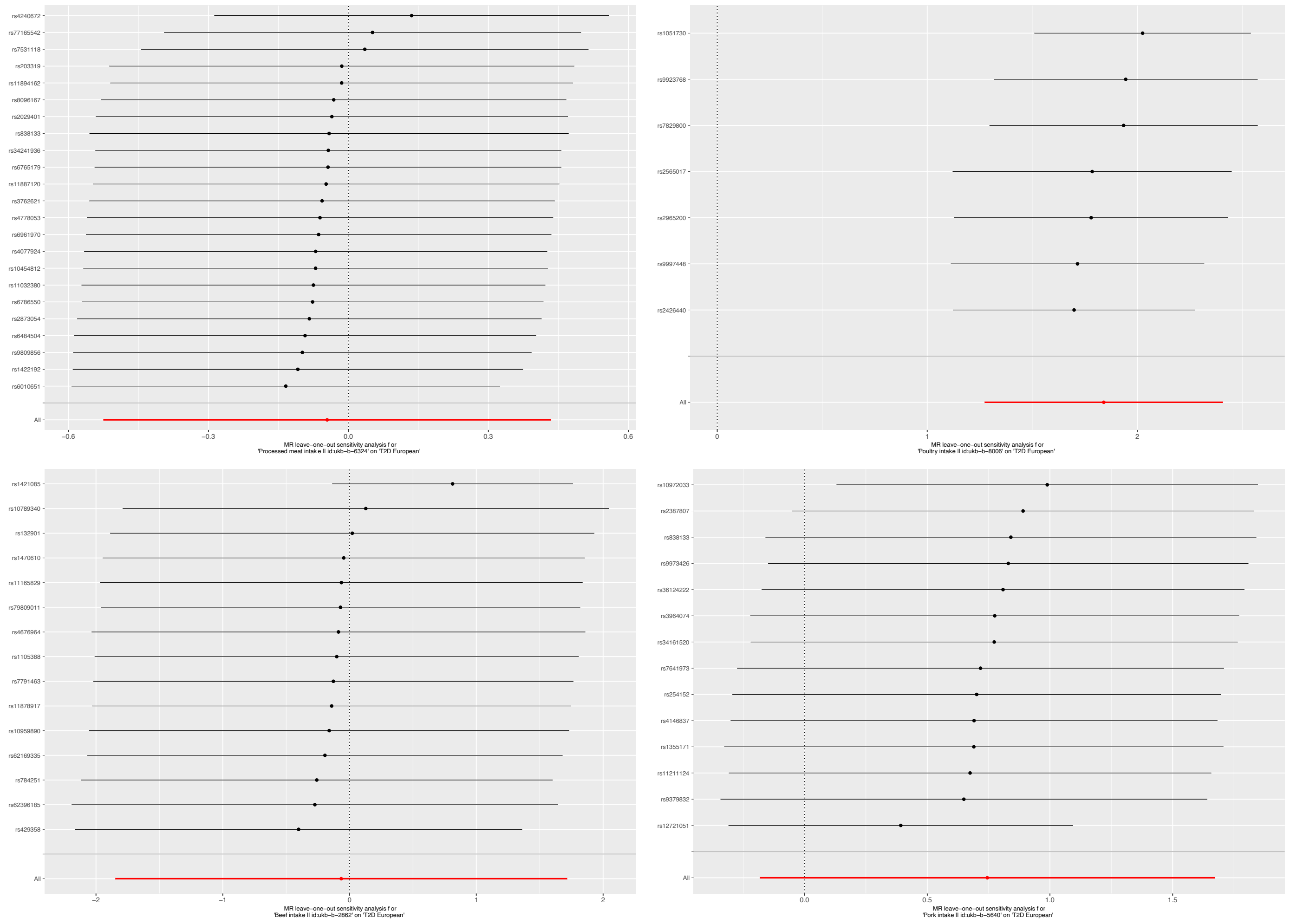
rs4240672
rs77165542
rs7531118
rs203319
rs11894162
rs8096167
rs2029401
rs838133
rs34241936
rs6765179
rs11887120
rs3762621
rs4778053
rs6961970
rs4077924
rs10454812
rs11032380
rs6786550
rs2873054
rs6484504
rs9809856
rs1422192
rs6010651
All
−0.6
−0.3
0.0
0.3
0.6
MR leave−one−out sensitivity analysis f or
'Processed meat intak e || id:ukb−b−6324' on 'T2D European'
rs1051730
rs9923768
rs7829800
rs2565017
rs2965200
rs9997448
rs2426440
All
0
1
2
MR leave−one−out sensitivity analysis f or
'Poultry intake || id:ukb−b−8006' on 'T2D European'
rs1421085
rs10789340
rs132901
rs1470610
rs11165829
rs79809011
rs4676964
rs1105388
rs7791463
rs11878917
rs10959890
rs62169335
rs784251
rs62396185
rs429358
All
−2
−1
0
1
2
MR leave−one−out sensitivity analysis f or
'Beef intake || id:ukb−b−2862' on 'T2D European'
rs10972033
rs2387807
rs838133
rs9973426
rs36124222
rs3964074
rs34161520
rs7641973
rs254152
rs4146837
rs1355171
rs11211124
rs9379832
rs12721051
All
0.0
0.5
1.0
1.5
MR leave−one−out sensitivity analysis f or
'Pork intake || id:ukb−b−5640' on 'T2D European'

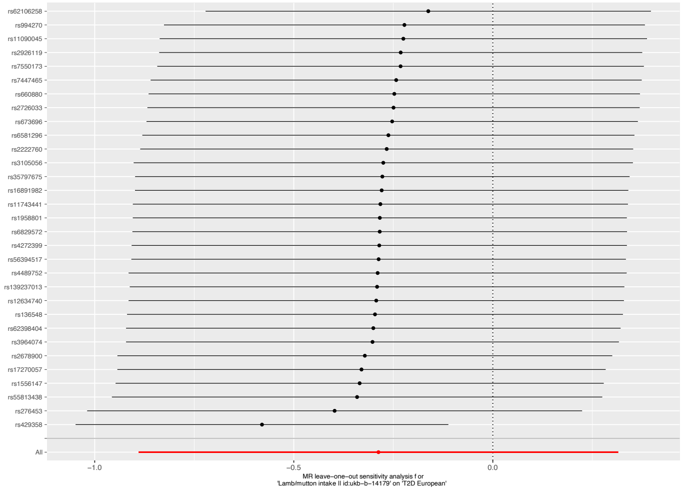
MR leave−one−out sensitivity analysis for
'Lamb/mutton intake || id:ukb−b−14179' on 'T2D European'
−1.0
−0.5
0.0
All

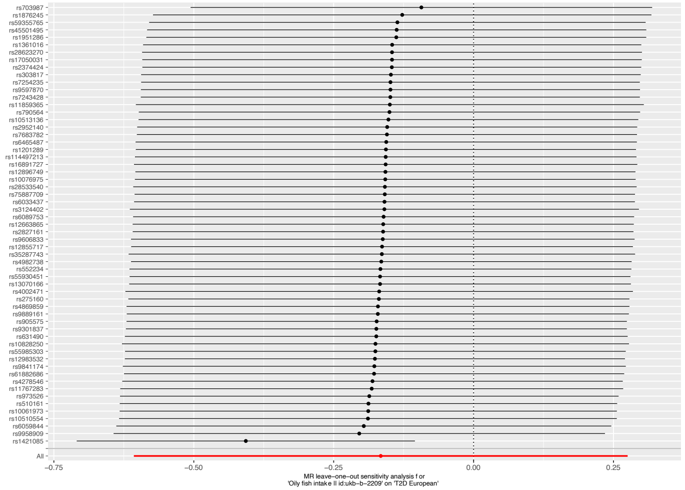
MR leave−one−out sensitivity analysis for
'Oily fish intake || id:ukb−b−2209' on 'T2D European'
−0.75
−0.50
−0.25
0.00
0.25
All

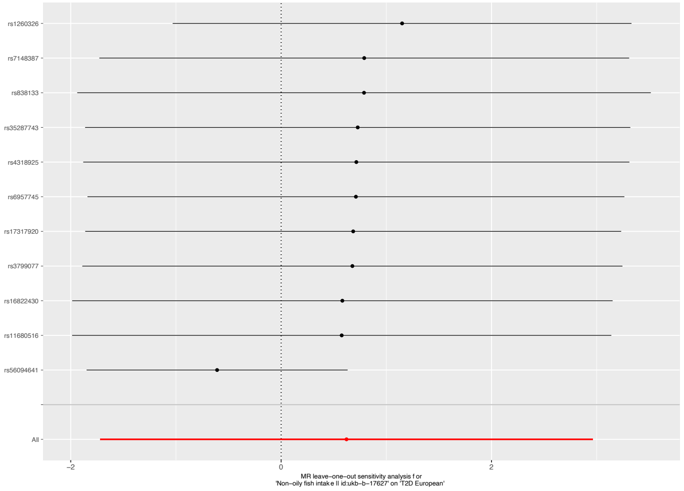
MR leave−one−out sensitivity analysis for
'Non−oily fish intake || id:ukb−b−17627' on 'T2D European'
−2
0
2
All

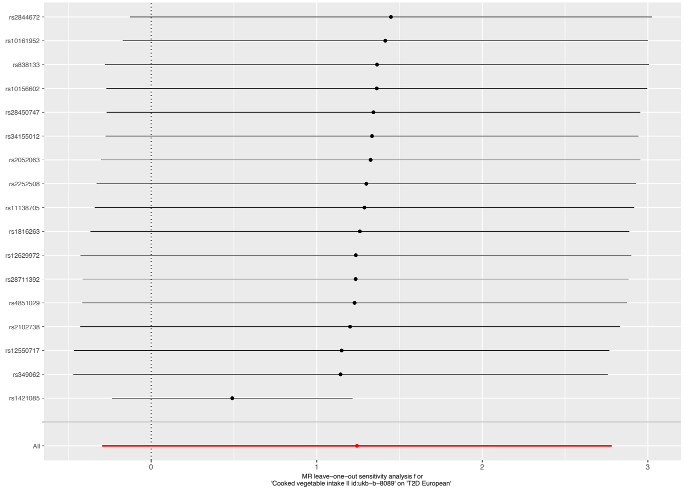
MR leave−one−out sensitivity analysis for
'Cooked vegetable intake || id:ukb−b−8089' on 'T2D European'
0
1
2
3
All

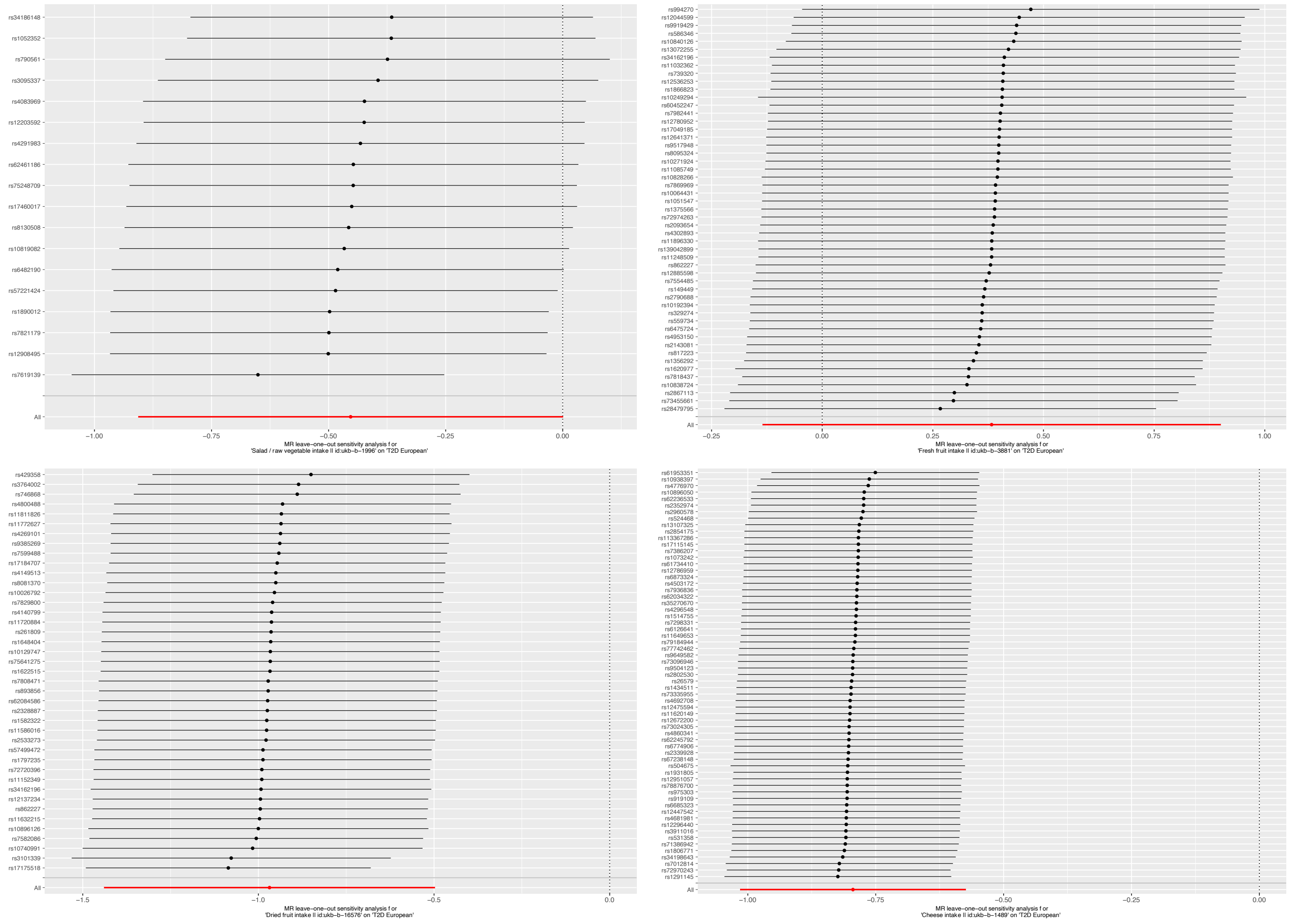
rs34186148
rs1052352
rs790561
rs3095337
rs4083969
rs12203592
rs4291983
rs62461186
rs75248709
rs17460017
rs8130508
rs10819082
rs6482190
rs57221424
rs1890012
rs7821179
rs12908495
rs7619139
All
−1.00 −0.75 −0.50 −0.25 0.00
MR leave−one−out sensitivity analysis f or
'Salad / raw vegetable intake II id:ukb−b−1996' on 'T2D European'
All
−0.25 0.00 0.25 0.50 0.75 1.00
MR leave−one−out sensitivity analysis f or
'Fresh fruit intake II id:ukb−b−3881' on 'T2D European'
All
−1.5 −1.0 −0.5 0.0
MR leave−one−out sensitivity analysis f or
'Dried fruit intake II id:ukb−b−16576' on 'T2D European'
All
−1.00 −0.75 −0.50 −0.25 0.00
MR leave−one−out sensitivity analysis f or
'Cheese intake II id:ukb−b−1489' on 'T2D European'

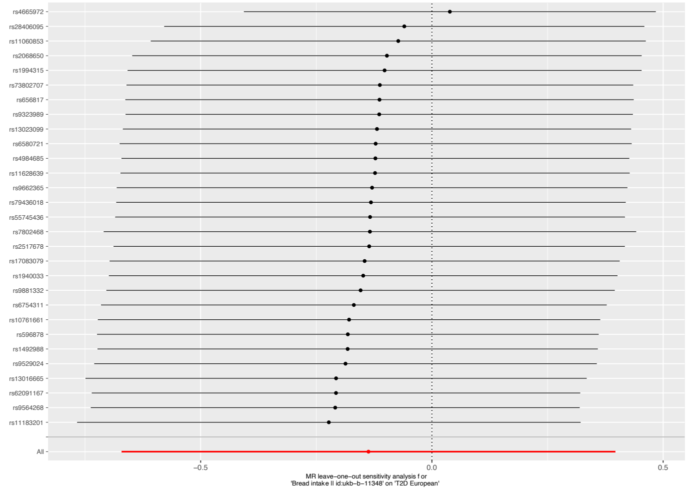
rs4665972
rs28406095
rs11060853
rs2068650
rs1994315
rs73802707
rs656817
rs9323989
rs13023099
rs6580721
rs4984685
rs11628639
rs9662365
rs79436018
rs55745436
rs7802468
rs2517678
rs17083079
rs1940033
rs9881332
rs6754311
rs10761661
rs596878
rs1492988
rs9529024
rs13016665
rs62091167
rs9564268
rs11183201
All
−0.5
0.0
0.5
MR leave−one−out sensitivity analysis f or
'Bread intake II id:ukb−b−11348' on 'T2D European'

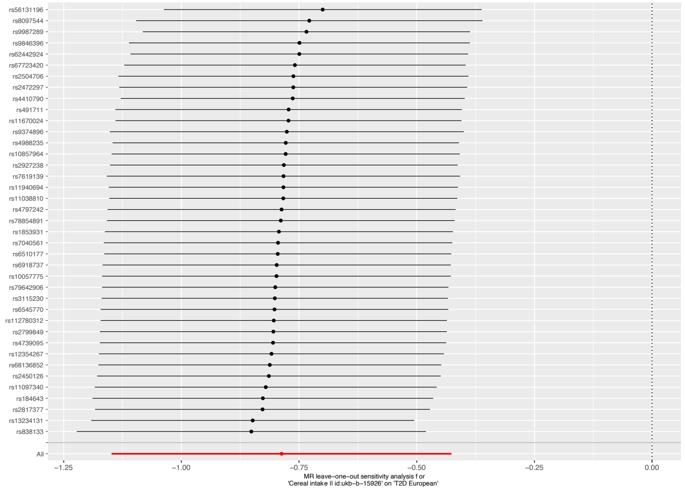
rs56131196
rs8097544
rs9987289
rs9846396
rs62442924
rs67723420
rs2504706
rs2472297
rs4410790
rs491711
rs11670024
rs9374896
rs4988235
rs10857964
rs2927238
rs7619139
rs11940694
rs11038810
rs4797242
rs78854891
rs1853931
rs7040561
rs6510177
rs6918737
rs10057775
rs79642906
rs3115230
rs6545770
rs112780312
rs2799849
rs4739095
rs12354267
rs68136852
rs2450126
rs11097340
rs184643
rs2817377
rs13234131
rs838133
All
−1.25
−1.00
−0.75
−0.50
−0.25
0.00
MR leave−one−out sensitivity analysis f or
'Cereal intake II id:ukb−b−15926' on 'T2D European'

Figure S1.3 Forest plot of Mendelian randomization effect size for dietary habits on T2D

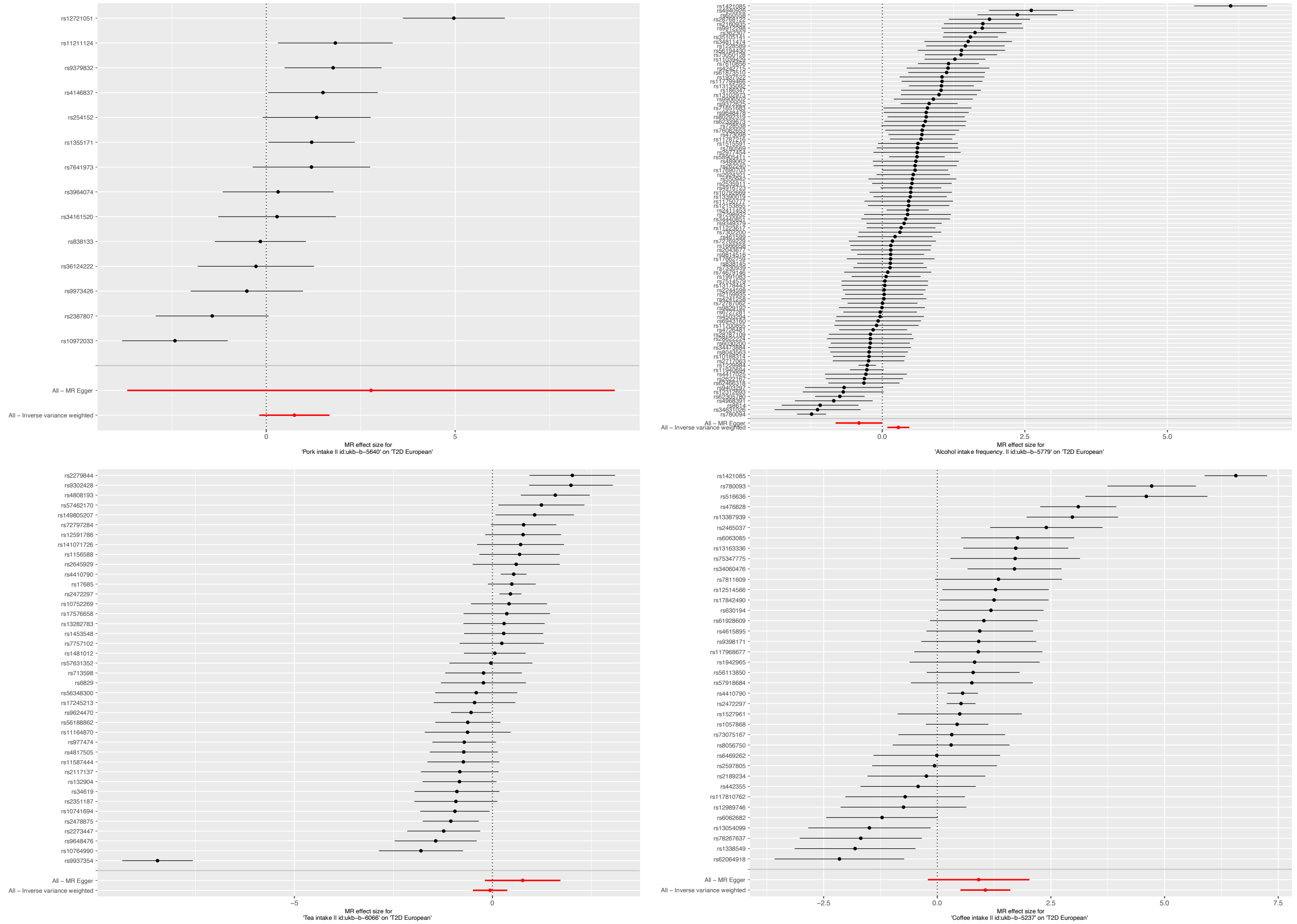

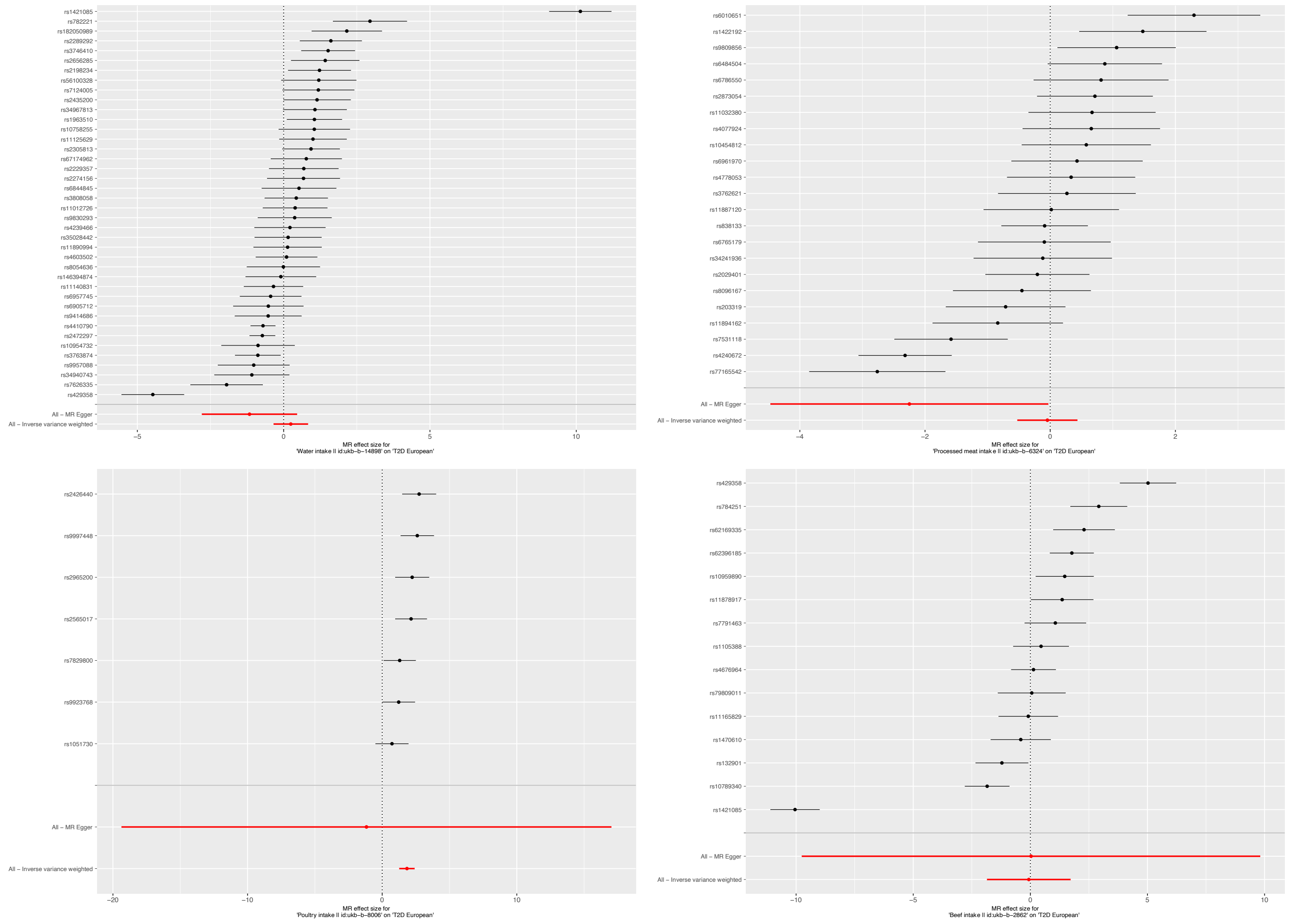

rs1421085
rs782221
rs182050989
rs2289292
rs3746410
rs2656285
rs2198234
rs56100328
rs7124005
rs2435200
rs34967813
rs1963510
rs10758255
rs11125629
rs2305813
rs67174962
rs2229357
rs2274156
rs6844845
rs3808058
rs11012726
rs9830293
rs4239466
rs35028442
rs11890994
rs4603502
rs8054636
rs146394874
rs11140831
rs6957745
rs6905712
rs9414686
rs4410790
rs2472297
rs10954732
rs3763874
rs9957088
rs34940743
rs7626335
rs429358
All – MR Egger
All – Inverse variance weighted
−5
0
5
10
MR effect size for
'Water intake II id:ukb−b−14898' on 'T2D European'
rs6010651
rs1422192
rs9809856
rs6484504
rs6786550
rs2873054
rs11032380
rs4077924
rs10454812
rs6961970
rs4778053
rs3762621
rs11887120
rs838133
rs6765179
rs34241936
rs2029401
rs8096167
rs203319
rs11894162
rs7531118
rs4240672
rs77165542
All – MR Egger
All – Inverse variance weighted
−4
−2
0
2
MR effect size for
'Processed meat intak e II id:ukb−b−6324' on 'T2D European'
rs2426440
rs9997448
rs2965200
rs2565017
rs7829800
rs9923768
rs1051730
All – MR Egger
All – Inverse variance weighted
−20
−10
0
10
MR effect size for
'Poultry intake II id:ukb−b−8006' on 'T2D European'
rs429358
rs784251
rs62169335
rs62396185
rs10959890
rs11878917
rs7791463
rs1105388
rs4676964
rs79809011
rs11165829
rs1470610
rs132901
rs10789340
rs1421085
All – MR Egger
All – Inverse variance weighted
−10
−5
0
5
10
MR effect size for
'Beef intake II id:ukb−b−2862' on 'T2D European'

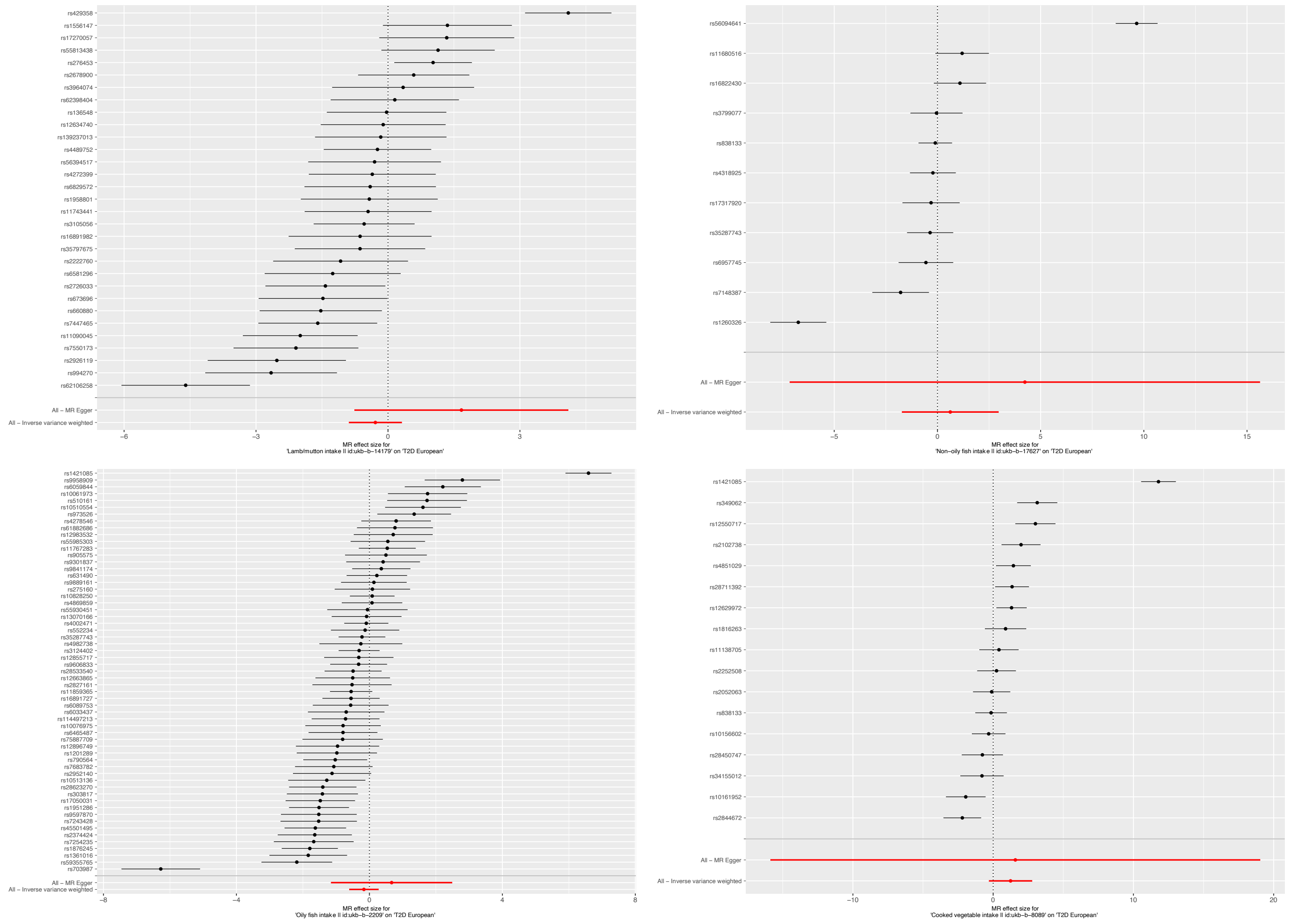
rs429358
rs1556147
rs17270057
rs55813438
rs276453
rs2678900
rs3964074
rs62398404
rs136548
rs12634740
rs139237013
rs4489752
rs56394517
rs4272399
rs6829572
rs1958801
rs11743441
rs3105056
rs16891982
rs35797675
rs2222760
rs6581296
rs2726033
rs673696
rs660880
rs7447465
rs11090045
rs7550173
rs2926119
rs994270
rs62106258
All – MR Egger
All – Inverse variance weighted
−6
−3
0
3
MR effect size for
'Lamb/mutton intake || id:ukb−b−14179' on 'T2D European'
rs56094641
rs11680516
rs16822430
rs3799077
rs838133
rs4318925
rs17317920
rs35287743
rs6957745
rs7148387
rs1260326
All – MR Egger
All – Inverse variance weighted
−5
0
5
10
15
MR effect size for
'Non−oily fish intake || id:ukb−b−17627' on 'T2D European'
rs1421085
rs9958909
rs6059844
rs10061973
rs510161
rs10510554
rs973526
rs4278546
rs61882686
rs12983532
rs55985303
rs11767283
rs905575
rs9301837
rs9841174
rs631490
rs9889161
rs275160
rs10828250
rs4869859
rs55930451
rs13070166
rs4002471
rs552234
rs35287743
rs4982738
rs3124402
rs12855717
rs9606833
rs28533540
rs12663865
rs2827161
rs11859365
rs16891727
rs6089753
rs6033437
rs114497213
rs10076975
rs6465487
rs75887709
rs12896749
rs1201289
rs790564
rs7683782
rs2952140
rs10513136
rs28623270
rs303817
rs17050031
rs1951286
rs9597870
rs7243428
rs45501495
rs2374424
rs7254235
rs1876245
rs1361016
rs59355765
rs703987
All – MR Egger
All – Inverse variance weighted
−8
−4
0
4
8
MR effect size for
'Oily fish intake || id:ukb−b−2209' on 'T2D European'
rs1421085
rs349062
rs12550717
rs2102738
rs4851029
rs28711392
rs12629972
rs1816263
rs11138705
rs2252508
rs2052063
rs838133
rs10156602
rs28450747
rs34155012
rs10161952
rs2844672
All – MR Egger
All – Inverse variance weighted
−10
0
10
20
MR effect size for
'Cooked vegetable intake || id:ukb−b−8089' on 'T2D European'

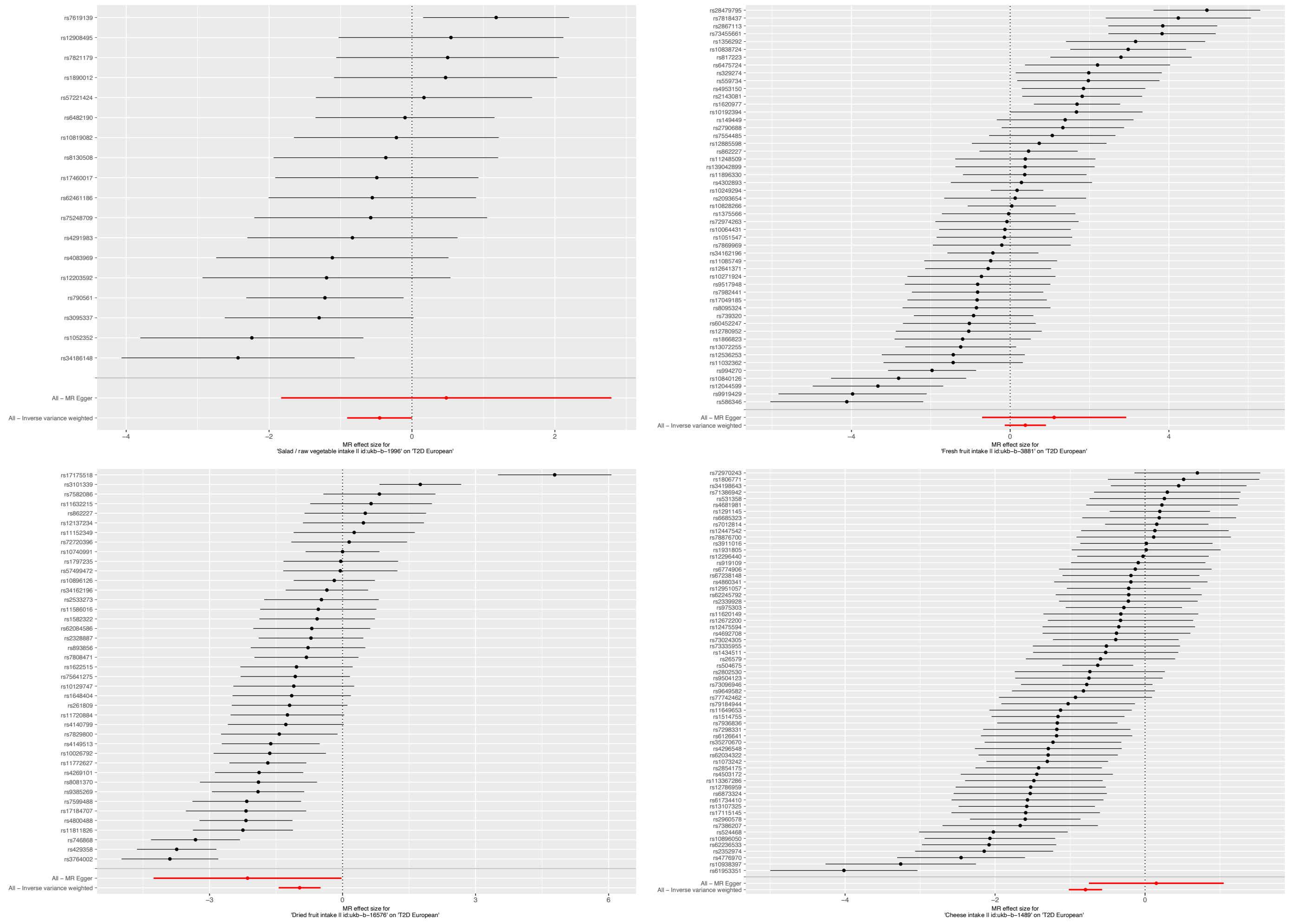
All – MR Egger
All – Inverse variance weighted
MR effect size for
'Salad / raw vegetable intake || id:ukb-b-1996' on 'T2D European'
MR effect size for
'Fresh fruit intake || id:ukb-b-3881' on 'T2D European'
MR effect size for
'Dried fruit intake || id:ukb-b-16576' on 'T2D European'
MR effect size for
'Cheese intake || id:ukb-b-1489' on 'T2D European'

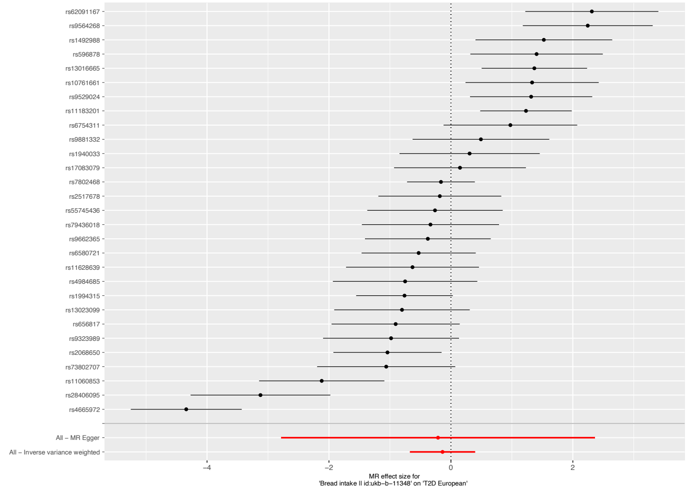
rs62091167
rs9564268
rs1492988
rs596878
rs13016665
rs10761661
rs9529024
rs11183201
rs6754311
rs9881332
rs1940033
rs17083079
rs7802468
rs2517678
rs55745436
rs79436018
rs9662365
rs6580721
rs11628639
rs4984685
rs1994315
rs13023099
rs656817
rs9323989
rs2068650
rs73802707
rs11060853
rs28406095
rs4665972
All – MR Egger
All – Inverse variance weighted
−4
−2
0
2
MR effect size for
'Bread intake II id:ukb−b−11348' on 'T2D European'

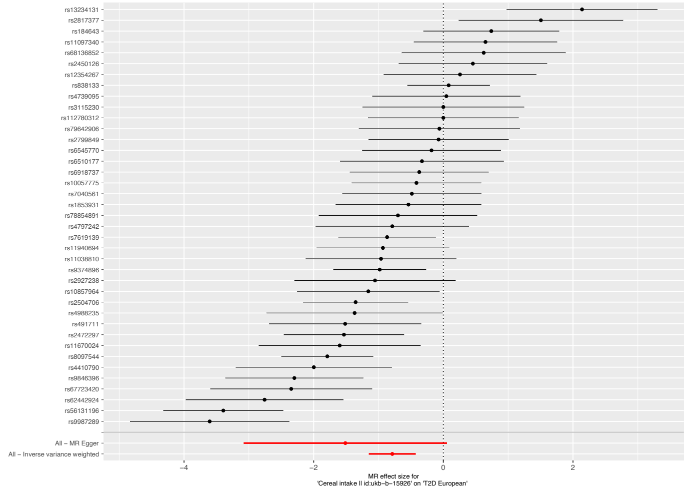
rs13234131
rs2817377
rs184643
rs11097340
rs68136852
rs2450126
rs12354267
rs838133
rs4739095
rs3115230
rs112780312
rs79642906
rs2799849
rs6545770
rs6510177
rs6918737
rs10057775
rs7040561
rs1853931
rs78854891
rs4797242
rs7619139
rs11940694
rs11038810
rs9374896
rs2927238
rs10857964
rs2504706
rs4988235
rs491711
rs2472297
rs11670024
rs8097544
rs4410790
rs9846396
rs67723420
rs62442924
rs56131196
rs9987289
All – MR Egger
All – Inverse variance weighted
−4
−2
0
2
MR effect size for
'Cereal intake II id:ukb−b−15926' on 'T2D European'

Figure S1.4 Funnel plot of Mendelian randomization effect size for dietary habits on T2D

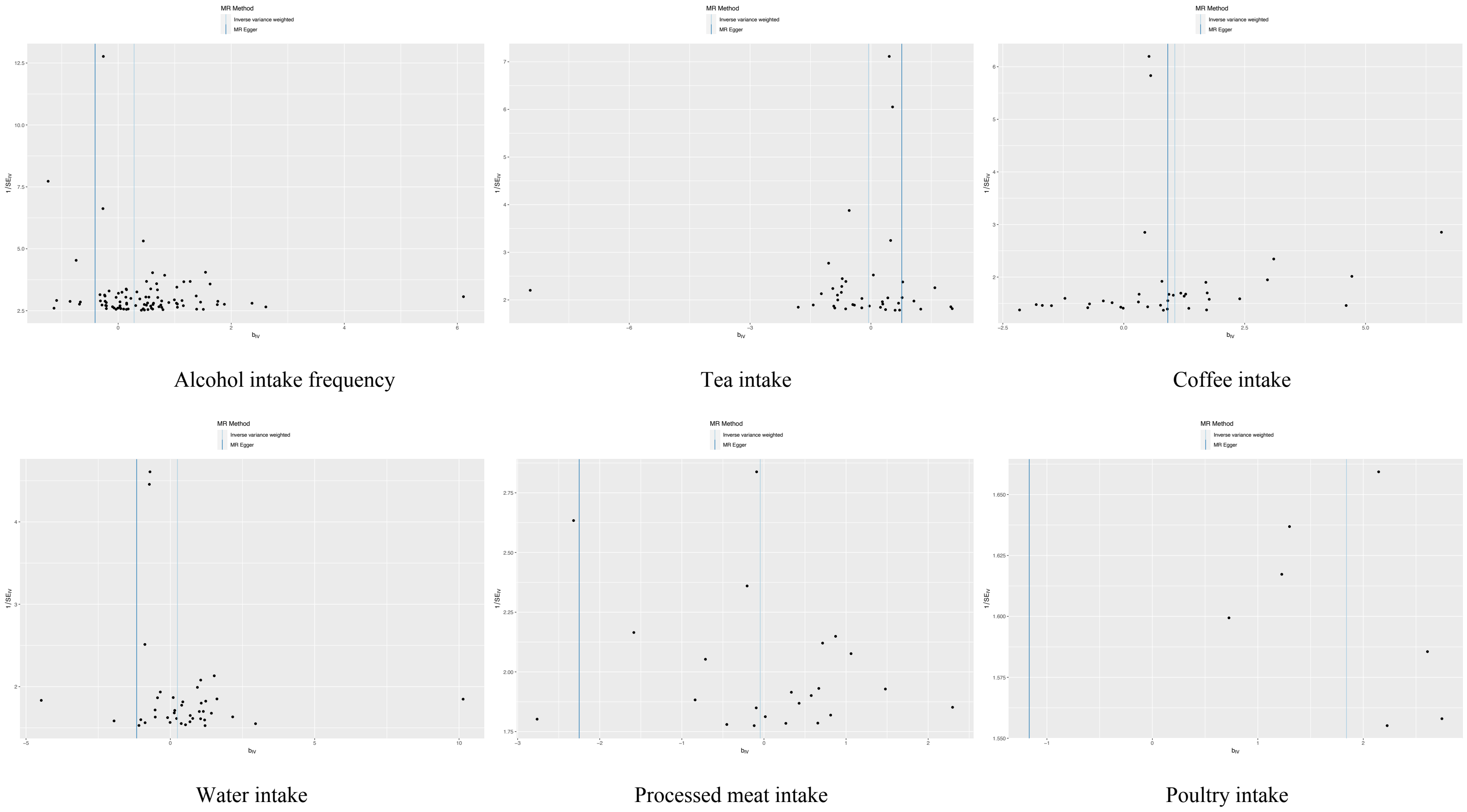

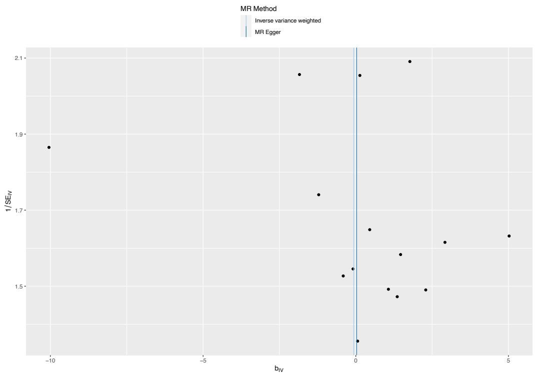

Beef intake

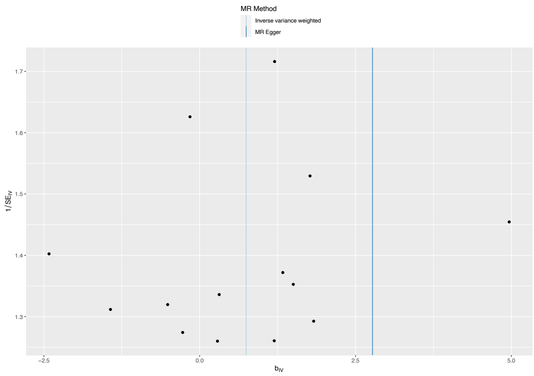

Pork intake

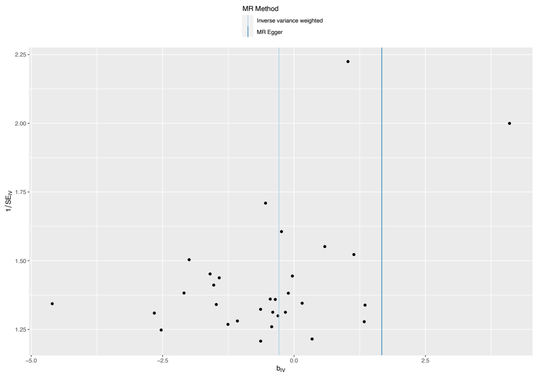

Lamb/mutton intake

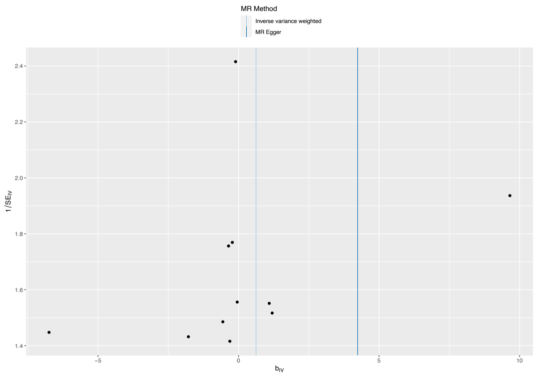

Non-oily fish intake

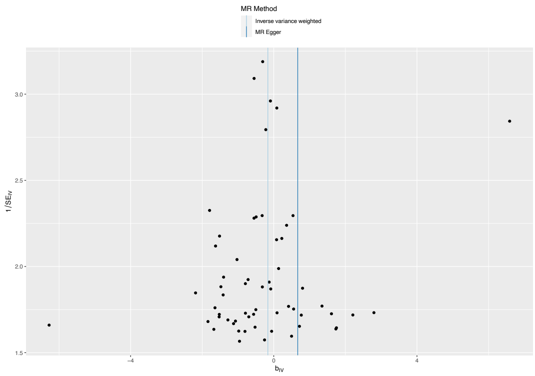

Oily fish intake

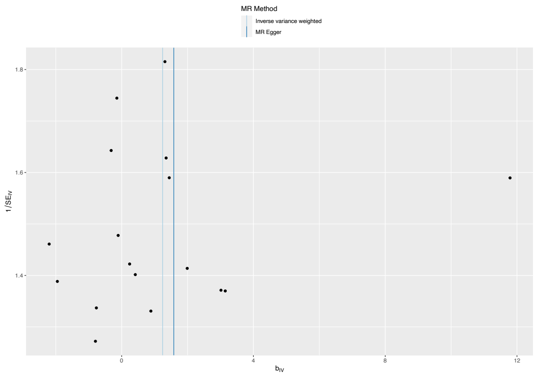

Cooked vegetable intake

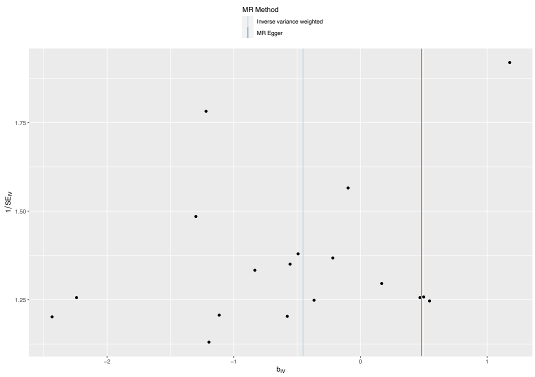

Salad/raw vegetable intake

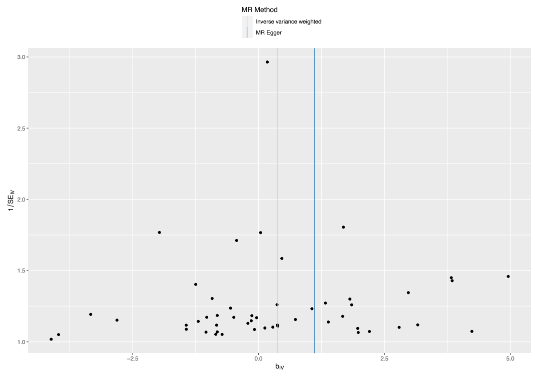

Fresh fruit intake

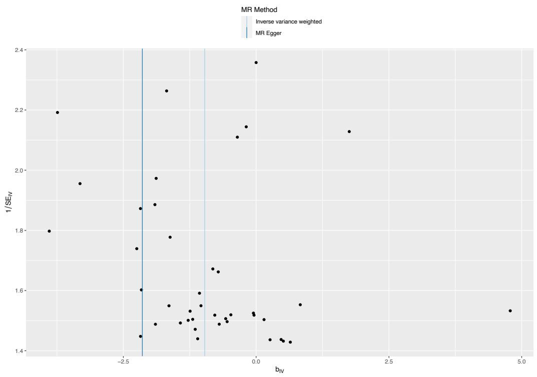

Dried fruit intake

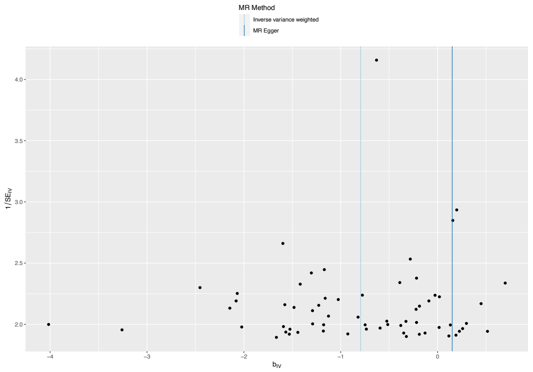

Cheese intake

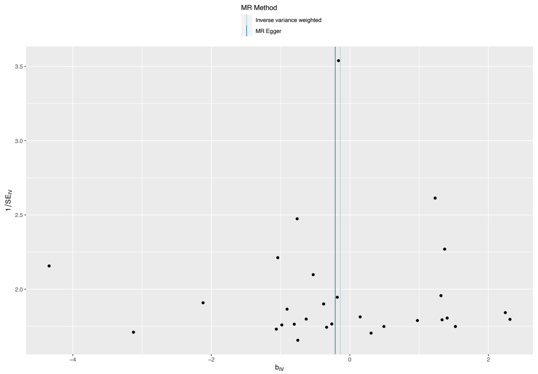

Bread intake

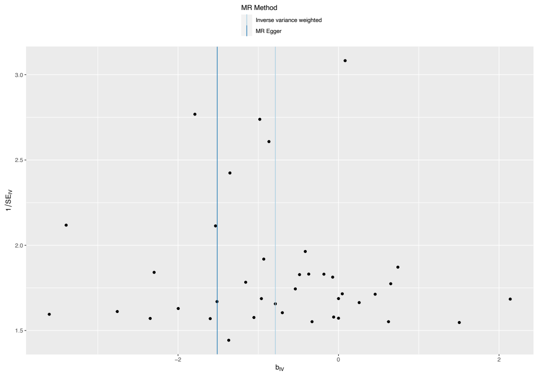

Cereal intake
